# Spatially Explicit Estimates of Prey Consumption Reveal a New Krill Predator in the Southern Ocean

**DOI:** 10.1371/journal.pone.0086452

**Published:** 2014-01-24

**Authors:** Andrea Walters, Mary-Anne Lea, John van den Hoff, Iain C. Field, Patti Virtue, Sergei Sokolov, Matt H. Pinkerton, Mark A. Hindell

**Affiliations:** 1 Institute for Marine and Antarctic Studies, University of Tasmania, Hobart, Tasmania, Australia; 2 Antarctic Climate and Ecosystems Cooperative Research Centre, Hobart, Tasmania, Australia; 3 Australian Antarctic Division, Kingston, Tasmania, Australia; 4 Marine Mammal Research Group, Department of Environment and Geography, Macquarie University, Sydney, New South Wales, Australia; 5 Commonwealth Scientific and Industrial Research Organisation Marine and Atmospheric Research, Hobart, Tasmania, Australia; 6 National Institute of Water and Atmospheric Research Ltd, Kilbernie, Wellington, New Zealand; Institut Pluridisciplinaire Hubert Curien, France

## Abstract

Development in foraging behaviour and dietary intake of many vertebrates are age-structured. Differences in feeding ecology may correlate with ontogenetic shifts in dispersal patterns, and therefore affect foraging habitat and resource utilization. Such life-history traits have important implications in interpreting tropho-dynamic linkages. Stable isotope ratios in the whiskers of sub-yearling southern elephant seals (*Mirounga leonina*; n = 12) were used, in conjunction with satellite telemetry and environmental data, to examine their foraging habitat and diet during their first foraging migration. The trophic position of seals from Macquarie Island (54°30′S, 158°57′E) was estimated using stable carbon (δ^1^ ^3^C) and nitrogen (δ^15^N) ratios along the length of the whisker, which provided a temporal record of prey intake. Satellite-relayed data loggers provided details on seal movement patterns, which were related to isotopic concentrations along the whisker. Animals fed in waters south of the Polar Front (>60°S) or within Commission for the Conservation of Antarctic Marine Living Resources (CCAMLR) Statistical Subareas 88.1 and 88.2, as indicated by both their depleted δ^1^ ^3^C (<−20‰) values, and tracking data. They predominantly exploited varying proportions of mesopelagic fish and squid, and crustaceans, such as euphausiids, which have not been reported as a prey item for this species. Comparison of isotopic data between sub-yearlings, and 1, 2 and 3 yr olds indicated that sub-yearlings, limited by their size, dive capabilities and prey capture skills to feeding higher in the water column, fed at a lower trophic level than older seals. This is consistent with the consumption of euphausiids and most probably, Antarctic krill (*Euphausia superba*), which constitute an abundant, easily accessible source of prey in water masses used by this age class of seals. Isotopic assessment and concurrent tracking of seals are successfully used here to identify ontogenetic shifts in broad-scale foraging habitat use and diet preferences in a highly migratory predator.

## Introduction

The interplay between the physical and biological regimes of the Southern Ocean [1] dictates the dispersal, foraging habitats and diet of higher order predators [2]. Information on diet is fundamental to better understand the diversity of linkages within Southern Ocean marine ecosystems and the response of higher order predators to large-scale ecosystem change and other anthropogenic activities such as commercial fishing [3]. Due to their marine existence, the dietary study of marine mammals is one of the most challenging of any vertebrate taxon [4]. Moreover, marine mammal species often exhibit ontogenetic shifts in dispersal patterns, foraging habitat and resource utilisation [5]. Such life-history traits have important individual and population level implications and must be taken into account when assessing the diet and trophic interactions of a species within an ecosystem.

Natal dispersal is a fundamental, but poorly understood, demographic parameter [6], particularly amongst vertebrate marine predators [7]. The mechanism which governs this phenomenon is largely unknown [8], although intra-specific competition for resources (*e.g.* food, space and mates) is one of the main hypothesises advanced to explain natal dispersal in the life history of most species [9].

The southern elephant seal (*Mirounga leonina*) is such an example of a polar species which exhibits an extreme natal dispersal strategy [10]. At weaning, adult female seals depart for remote feeding grounds, leaving pups to spend another three to eight weeks ashore before they too depart natal colonies [11], [12]. The lack of maternal input into dispersal strategies means the likelihood of these young animals foraging successfully, in an unfamiliar ocean environment, is largely dependent on chance. Consequently, this may contribute to the relatively high first year mortality in this species [13].

Surviving seals disperse over many thousands of kilometres [14] to access different prey communities [15], and seem to develop site fidelity to areas known to previously provide good feeding [16]. This behaviour is also noted for their northern counterpart, the northern elephant seal (*M. angustirostris*) [17]. Dispersal patterns may correlate with increased intra-specific competition for resources, physiological capabilities (*e.g.* related to size, sex, diving capacity), familiarity with habitat, temporal shifts in haul-out behaviour and reduced mortality risks of extensive movement [5], [14], [18]. Adult females from Macquarie Island, constrained by breeding requirements, make directed movements south of the major Antarctic Circumpolar Current (ACC) fronts to feed over the East Antarctic continental shelf before the winter sea ice makes this habitat inaccessible [19]. In contrast, younger seals, constrained by physiological capabilities [20], make less directed travel, predominantly north of the southern limits of the ACC, closer to their natal island [10], [14], [21].

What is known about the diet of this species stems largely from studies of stomach contents and faecal analysis [5], [22], [23], but interpretation is impeded by the wide separation between feeding and haul-out sites [24]. Stable isotope analysis, which assesses ratios of carbon (^13^C/^12^C; δ^13^C) and nitrogen (^15^N/^14^N; δ^15^N) isotopes in various body tissues, is being increasingly used to study the foraging habitat and trophic position of highly migratory animals [25], [26], [27], [28], [29] as it can yield a data time-series derived from assimilated, and not just ingested food [30].

Carbon (*e.g.* ^13^C) concentrations change by only ∼0.8 to 2‰ per trophic level, reflecting the source of carbon at the base of the food chain [31], [32] and thus consumer’s foraging habitat. Nitrogen (*e.g.* ^15^N) concentrations in consumer tissues typically increase at ∼3‰ per trophic level [33], [34] rendering them particularly useful in estimating prey trophic position [35]. As whiskers are keratin-based tissues, which are metabolically inert after synthesis [26], they approximate a time-line of stable isotope values derived from food sources, with the tip of the whisker representing the oldest growth, and the root the most recent growth [36].

In this study, stable isotope and satellite telemetry for consumers and corresponding environmental data are combined to quantify the feeding habits and trophic position of sub-yearling elephant seals in relation to habitat during their first feeding migration from Macquarie Island. The specific aims of this study were to determine: 1) the growth rates of whiskers of sub-yearlings in the six months after weaning, and 2) the trophic position (*i.e.* δ^13^C and δ^15^N) of seals in relation to foraging location, water mass type and seal age.

## Materials and Methods

### Ethics Statement

Animals in this study were cared for in accordance with the guidelines of the University of Tasmania Animals Ethics Committee that approved the fieldwork (Permit no. A0006738, M. Hindell).

### Data Collection

#### Seal whiskers

Facial vibrissae (whiskers) were collected from southern elephant seal pups equipped with Satellite Relayed Data Loggers (SRDLs, Sea Mammal Research Unit, St Andrews, UK), which consisted of a data logger interfaced to a 0.5-W Argos radio frequency unit [37]. Satellite Relayed Data Loggers were fitted to pups during their post-weaning fast at Macquarie Island (54°30′S, 158°57′E) in December 1995 (n = 6) and 1999 (n = 6). A six hour record was summarized and transmitted. One whisker was collected from each individual at SRDL deployment (pre-trip whisker; 1995 only, n = 5) and a second whisker was collected when the SRDL was retrieved approximately 4 to 7 months later (post-trip whisker; n = 12; Table 1). All animals were sampled within 7 days of their return (mean = 3.1±2.5 days, n = 12). Whiskers were not plucked but cut as close to the face as possible from the same location on the left hand side of the muzzle (C.R. McMahon, Personal communication). Details of the capture, handling and attachment of telemetry devices to study animals are provided elsewhere [38], [39], [40].

**Table 1:** Morphometric, tag deployment and tracking details for 12 weaned southern elephant seals from Macquarie Island, including the number of days (mean±SD) spent in transit and Area Restricted Search (ARS) by seals.

Seal IDNo.	Sex	WeaningMass (Kg)	DeploymentMass (Kg)	Transit duration(days)	ARS duration(days)	Total duration(days)
**1995/1996**						
**J226**	F	78.0	62.0	47.0	70.0	126.0
**J263**	M	143.0	107.0	45.0	86.0	149.0
**J373**	F	92.0	73.0	56.0	66.0	130.0
**J375**	F	89.0	68.0	12.0	62.0	146.0
**J492**	F	88.0	62.0	50.0	72.0	148.0
**J503**	M	92.0	66.0	46.0	81.0	137.0
**Mean**		97.0±23.1	73.0±17.2	42.7±15.5	72.8±9.0	139.3±9.8
**1999/2000**						
**T719**	F	195.0	123.0	42.0	108.0	178.0
**T825**	F	104.0	96.0	57.0	105.0	182.0
**T839**	F	101.0	71.0	56.0	79.0	139.0
**T867**	F	90.0	62.0	50.0	95.0	179.0
**T875**	F	85.0	60.0	76.0	62.0	155.0
**T887**	F	93.0	70.0	63.0	94.0	169.0
**Mean**		111.3±41.6	80.3±24.5	57.3±11.6	90.5±17.3	167.0±16.9
**Overall mean**		104.2±32.9	76.7±20.5	50.0±15.1	81.7±16.1	153.2±19.5

#### Prey specimens

In the absence of available prey stable isotope data for this region of the Southern Ocean we used published and unpublished values corresponding to latitudinal ranges similar to this population of juvenile southern elephant seals, although outside the current foraging range. We have assumed that despite the geographic disparity, the prey isotope values will be broadly consistent with those within the seals foraging areas. Mid-latitude (<55°S) specimens of fish (*Protomyctophum tenisoni*, n = 8; *Electrona antarctica*, n = 10; *Gymnoscopelus piabilis*, n = 2; *G. nicholsi*, n = 10 and *G. fraseri*, n = 8) were collected by the RV *La Curieuse* during bathypelagic trawls, to the northeast of the Kerguelen Archipelago (49°07′S, 70°45′E) in June 1998 (see [41]). The samples were collected at night using an IYGPT net (International Young Gadoid Pelagic Trawl net; opening: 12×7 m) with a 10 mm mesh size in the cod end [42], and were sorted on deck and frozen. Lower beaks of two squid species (*Martialia hyadesi*, n = 66 and *Histioteuthis eltaninae*, n = 71) were obtained from the stomach contents of juvenile southern elephants seals (one, two and three year olds) during their annual haul-out periods as they returned ashore at Macquarie Island (54°30′S, 158°57′E), from November 1997 and December 2000 (Hughes, A.R. unpubl. data). Details of the capture, handling and stomach lavaging of study animals are provided elsewhere [5], [43], [44]. The filtered stomach contents were stored in 70% ethanol.

High latitude (>60°S) specimens of fish (*E. antarctica*, n = 10), euphausiids (*Euphausia triacantha*, n = 10), hyperiid amphipods (*Themisto gaudichaudii*, n = 7) and squid (*Bathyteuthis abysicola*, n = 2 and *Psychroteuthis glacialis*, n = 3) were collected by the Japanese TRV *Umitaka Maru* using pelagic trawls in the Dumont d’Urville Sea, ranging from Terre Adélie to the Mertz Glacier tongue, in George V Land (61°45′ to 67°30′S, 140° to 143°E) as part of the Collaborative East Antarctic Marine Census (CEAMARC) in January/February 2008 [45], [46]. Samples were collected at night and day using an IYGPT net (opening: 5.5×12 m) with a mesh of 100 mm in the front, then tapering through 80 mm to 40 mm to 20 mm to 10 mm mesh in the cod end and were sorted on deck and frozen. Samples were stored at −80°C until analysis.

### Foraging Habitat

We fitted a first-difference correlated random walk switching (DCRWS) model [47] incorporating Argos error to elephant seal satellite location data originating and terminating at Macquarie Island (n = 14). Using 360 minute time step intervals, the model indexed movement parameters (differences in latitude and longitude between consecutive positions along the track) according to two behavioural modes [48]; transit and Area Restricted Search (ARS) modes. Area Restricted Search corresponded to periods of reduced travel speed and increased turning rate (parameter estimates between 1.8 and 2.0), which are more likely to be associated with foraging movements as opposed to transit movement (parameter estimates between 1.0 and 1.2) [48]. Locations which did not fit these criteria (*i.e.* parameter estimates between 1.2 and 1.8; 14.2% of all locations at sea) were discarded. The methodology used to fit the model to elephant seal location data is described in detail in Jonsen et al. [49].

Using the ARS locations, we calculated the proportion (%) of time spent by the seals in defined ACC Inter-Frontal Zones (IFZs). To map the position of ACC fronts, we used 19 years (1992 to 2011) of weekly sea surface height (SSH) gradients. The approach used to identify fronts in SSH data is described in detail by Sokolov and Rintoul [50], [51], [52] and summarized briefly here.

To map fronts in the Southern Ocean, twelve SSH contours were used, as in Sokolov and Rintoul [51]. Of these, nine contours are associated with the ACC itself and three contours correspond to elevated SSH gradients associated with subtropical western boundary currents and their extension along the northern edge of the Southern Ocean. The ACC front positions inferred from satellite SSH maps were validated using independent data from Argo floats and high resolution hydrographic sections as described in detail in Sokolov and Rintoul [50], [51].

Each elephant seal satellite location was ascribed to an IFZ [50], [51], [53] defined as: (1) south of sBdy, (2) sBdy to SACCF-S, (3) SACCF-S to SACCF-N, (4) SACCF-N to PF-S, (5) PF-S to PF, (6) PF to PF-N, (7) PF-N to SAF-S, (8) SAF-S to SAF, (9) SAF to SAF-N, (10) SAF-N to SAZ, (11) SAZ to STZ-S, (12) STZ-S to STZ-N and (13) N STZ-N, where sBdy: southern Boundary Current; ACC: Antarctic Circumpolar Current; N: north; S: south; PF: Polar Front; SAF: sub-Antarctic Front; SAZ: sub-Antarctic Zone and STZ: sub-Tropical Zone.

These IFZs were summarised into seven distinct zones as follows: 1. S of SACCF-S (IFZ 1,2); 2. ACC to PF-S (IFZ 3,4); 3. PF (IFZ 5,6); 4. PF to SAF (IFZ 7); 5. SAF (IFZ 8,9); 6. SAF-N to SAZ (IFZ 10), and 7. SAZ to STZ-S (IFZ 11). Numbers in brackets correspond to IFZs above.

### Sample Preparation and Stable Isotope Analysis

#### Seal whiskers

The whiskers were cleaned with successive rinses in a 2∶1 chloroform:methanol solution, and then dried in an oven at 60°C for 72 hours. The twelve post-trip whiskers were weighed and sectioned into approximately 2 mm sections. The sections from each whisker were numbered sequentially, starting from the base, in order to track the temporal integration of isotope values along the length of the whisker.

#### Prey specimens

Isotopic analysis was performed on the white muscle of fish, the mantle and lower beaks of squid, and whole specimens of amphipods and euphausiids. Muscle tissues (fish and squid) and whole specimens were freeze dried and ground to fine powder before lipids were removed from all samples [54], and carbonates were removed from amphipod and euphausiid samples [55]. Different ratios of chitin (a ^15^N-depleted molecule) to protein are found in undarkened, darkening and darkened parts of squid beaks, with much more chitin in undarkened than in darkened parts [56], [57]. Consequently, the darkened wings of lower beaks are less impoverished in ^15^N relative to diet and were therefore used for stable isotope analysis. The lower beaks of squid were cleaned with successive rinses of distilled water, before the wing parts of beaks were cut away using scissors. Wings of lower beaks were then dried in oven at 60°C for a minimum of 16 hours and ground to fine powder. Relative abundance of ^13^C and ^15^N were determined using an Isoprime (Micromass, UK) continuous-flow isotope-ratio mass spectrometer. Results are reported using standard δ notation in parts per thousand (‰) relative to Pee Dee Belemnite (PDB) for δ^13^C and atmospheric N^2^ (Air) for δ^15^N as follows:
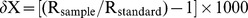
where δX is δ^13^C or δ^15^N, and R is the ratio of ^13^C/^12^C or ^15^N/^14^N.

Replicate measurements of internal laboratory standards (Alanine) indicate measurement errors <0.20 ‰ and <0.21 ‰ for δ^13^C and δ^15^N, respectively. Stable isotope analysis was performed by the Environmental Biology Group, Research School of Biological Sciences, Australian National University (ANU), Canberra, Australia.

### Whisker Growth Dynamics and Isotopic Values of Sub-yearlings

Southern elephant seals undergo a 24 day lactation period [58] in which post-partum pup growth is fuelled exclusively by energy from stored reserves in fasting mothers [12]. Isotope values along the length of a pre-trip whisker are therefore derived from *in-utero* development and post-partum, while isotopic values in the post-trip whisker reflect a shift from maternal investment to independent foraging. As pups mature, the process of weaning leads to a change in isotopic signal when the assimilation of carbon and nitrogen shifts to sources other than mother’s milk, such as free-ranging prey [59], [60], [61] and/or energy stores (fasting) post-weaning and pre-departure [11]. Thus, weaning essentially represents a change in trophic level from mother’s milk (higher trophic level) to free ranging prey (lower trophic level).

**Figure 1:**
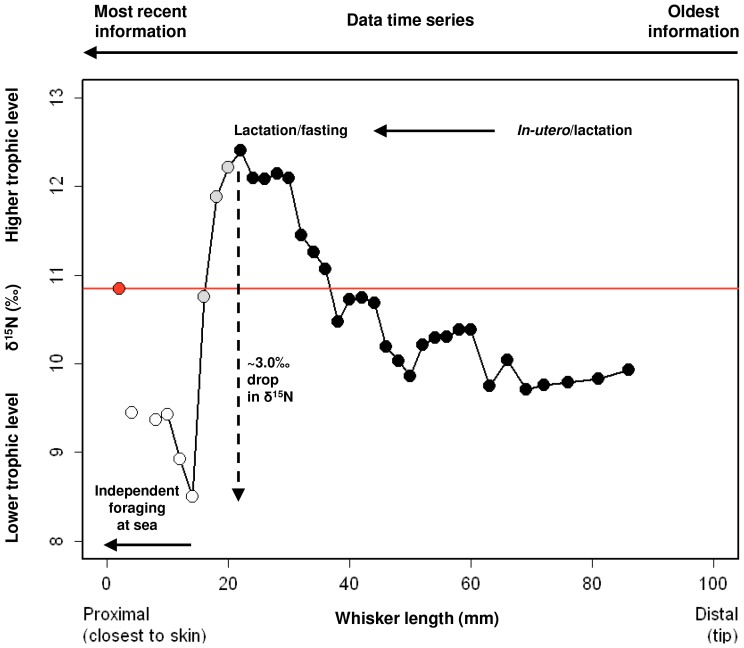
A schematic plot used to determine the shift to independent foraging along the post-trip whisker. We used stable nitrogen isotope values incorporated along the temporal span of the whisker as represented by the growth of the whisker from the distal (tip; oldest isotopic information) to proximal region (closest to the skin; most recent isotopic information). The red line indicates where the pre-trip basal segment (red symbol) intercepts δ^15^N values along the length of whisker. Solid arrows indicate the shift in food source along the temporal span from *in utero*/lactation to lactation/fasting (black symbols) to independent foraging at sea (open symbols). Dashed arrow indicates 3.9‰ drop in δ^15^N (equivalent to one trophic level ∼3.0‰; grey symbols). The first 14 mm of whisker represents independent foraging at sea.

To identify which part of the post-trip whisker reflected independent foraging at sea, we therefore compared δ^15^N values along the length of the post-trip whisker to the basal section of the pre-trip whisker (containing the isotopic signal of *in-utero* development and post-partum; red symbol, Fig. 1). The horizontal solid line indicates where the pre-trip basal segment intercepts the δ^15^N values along the length of the post-trip whisker (10.8‰). Note that the point of interception occurs during the drop of 3.9‰ in δ^15^N from 12.4 to 8.5‰, which we interpret as a trophic level shift from mother’s milk and/or fasting to independent foraging at sea. Once the lowest δ^15^N is reached (*i.e.* the transition is complete), we consider this and all subsequent samples to represent amino acids derived from independent foraging. The proportion (mm) of post-trip whisker that represents δ^15^N values incorporated during independent foraging at sea is 14 mm (Fig. 1).

### Variation in Habitat and Trophic Position (δ^13^C and δ^15^N) of Seals with the Location of Foraging

Within the Southern Ocean, there is a well defined geographical δ^13^C gradient in particulate organic matter (POM) surface waters, ranging from high δ^13^C values in warm subtropical waters in the north, to depleted values in cold Antarctic waters in the south [62], [63], [64], [65], [66]. This is subsequently transferred to higher levels within the food chain [67], [68]. In order to relate isotopic signatures to foraging habitat we therefore took into account the latitudinal gradient in tissue δ^13^C values of top predators in the Southern Ocean [67], [68] and the location of the major oceanographic frontal zones, *e.g.* SAF, PF and sBdy [53] of the Southern Ocean.

The greatest proportion of seal ARS locations occurring in a particular IFZ defined their habitat use. Isotopic signatures of elephant seals were then grouped according to habitat.

### Inferred Prey Consumption during the First Six Months

We used δ^13^C and δ^15^N values of marine organisms from mid- and high latitude Southern Ocean waters to infer the diet of elephant seals in relation to foraging habitat ([69], [70], [71], [72], this study; Table S1). For mid-latitude waters, we used a combination of pelagic fish (myctophids), squid and euphausiids from waters located around the PF in the Indian and Atlantic Ocean sectors of the Southern Ocean.

The beaks of two species of squid (*M. hyadesi* and *H. eltaniane*) contained in stomach lavage samples of returning juvenile Macquarie Island southern elephant seals were also examined (Hughes, A.R. unpubl. data). Cephalopod beak structure is species specific [73] and the lower beak rostral length (LRL) can be used to estimate mantle length and mass of squid from allometric data [74]. To assess the size of squid consumed by sub-yearlings we therefore used mean δ^13^C and δ^15^N values of small, medium and large LRL sized beaks according to species (for details see Table S1).

From high latitude waters (>60°S) north of Adélie and George V Land in the Indian Ocean sector, we used myctophids (*Electrona antarctica*), euphausiids (*Euphausia triacantha*) and amphipods (*Themisto gaudichaudii*) sampled in pelagic waters at depths of <500 m (62 to 65°30′S, 140 to 143°E); the deep-sea squid *Bathyteuthis abysicola*, sampled at depths of <1000 m (63°S, 140°E), and the glacial squid *Psychroteuthis glacialis*, sampled at depths of <200 m in high latitude waters (65°30′S, 140°E). Values of Antarctic krill (*Euphausia superba*) sorted from emperor penguin (*Aptenodytes forsteri*) regurgitates from Adélie Land [71], and of pelagic squid from the northern Ross Sea area in the Pacific Ocean sector, were also used [72].

We used whisker-specific isotopic fractionation values for δ^13^C and δ^15^N of 3.2‰ and 2.8‰, respectively, as obtained from a study of captive pinnipeds [30], since studies reporting the isotopic fractionation for δ^13^C and δ^15^N between diet and whiskers for wild populations of elephant seals or other pinniped species are absent. Correction factors of 3.2‰ and 2.8‰ were therefore applied to δ^13^C and δ^15^N values, respectively, when comparing seal values to the isotopic values of marine organisms. Cephalopod beaks are depleted in ^15^N, due to the presence of chitin (a ^15^N depleted molecule) and accordingly, contain lower δ^15^N values compared to the mantle (∼3.5‰) and buccal mass (∼2.6‰) soft tissue of cephalopods [56]. A correction factor of 3.5‰ was therefore applied to the δ^15^N values of *H. eltaninae* and *M. hyadesi* beaks prior to isotopic comparison with elephant seals and other marine organisms.

### Variation in Habitat and Trophic Position (δ^13^C and δ^15^N) of Seals with Age

To assess age-related shifts in trophic level and diet structure we compared the isotopic signatures in whiskers of sub-yearlings (n = 12; this study) to that of one (n = 5), two (n = 40) and three (n = 27) year old elephant seals sampled between 1999 and 2000 from Macquarie Island [75]. Isotopic signatures of one, two and three year old seals were derived from a single, randomly selected 2 mm section from each whisker.

### Statistical Analyses

We performed all statistical analysis using R version 2.15.0 [76]. To determine if the stable isotopic signatures (δ^13^C and δ^15^N) of migratory, sub-yearling elephant seal whiskers were influenced by sampling year or the location of foraging (as inferred by the proportion of ARS locations occurring in IFZs) we used multivariate analyses of variance (MANOVA) fitted with the MANOVA function in R. To determine if δ^13^C or δ^15^N values were influenced by sampling year or the location of foraging separately, we used a linear model fitted with the lm function in R, with whisker δ^13^C or δ^15^N values as the dependent variables, year and foraging location as factors and the two way interaction term. We used Analysis of Variance (ANOVA) along with Tukey’s Honestly Significant Difference (HSD) post-hoc analysis to indicate where response variables differed. Proportional data were arcsine transformed prior to statistical analysis.

Linear mixed-effects models were used to examine the effects of age class and sex on variation of stable isotope values. The dependent variable was either δ^13^C or δ^15^N, with sampling year as a random factor, and age and sex as fixed factors. If the distribution was significantly different from normality, the data were log-transformed and normality verified. Interactions between sampling year and dependent variables were examined. Effects of age and sex were, likewise, tested systematically in all analyses. We further assessed qualitative patterns of variation in habitat and trophic position through graphical examination of δ^13^C and δ^15^N values, respectively. We assessed significance for statistical tests at the 0.05 level. Mean values are given ± standard deviation (SD).

## Results

### Whisker Growth Dynamics and Isotopic Values of Sub-yearlings

Post-trip whiskers ranged in length from 42 to 144 mm (mean = 113.7±19.2 mm; n = 12; Table 2) with the number of segments per whisker ranging from 17 to 55 (mean = 33±9.4). Overall, a total of 551 sections were cut and analysed.

**Table 2:** Proportion of time spent (percentage) in Area Restricted Search (ARS) by seals in Inter-Frontal Zones (IFZs), including the Antarctic zone south of the southern Antarctic Circumpolar Current front-Southern Branch (S of SACCF-S), the ACC to Polar Front-Southern Branch (ACC to PF-S), the Polar Front (PF), the Polar Front to sub-Antarctic Front (PF to SAF) and the sub-Antarctic Front (SAF).

Seal IDNo.	Sex	Proportion of ARS locations occurring in IFZs (%)						Habitat group	
		S of SACCF-S	ACC to PF-S	PF	PF to SAF	SAF	Total	IFZ	Latitude (°S)
**1995/1996**									
**J226**	F	0.0	0.0	47.8	5.7	2.0	55.5 (Dec–Apr)	PF	62°41′
**J263**	M	0.0	28.6	23.1	6.0	0.0	57.7 (Dec–Mar)	ACC to PF-S	64°60′
**J373**	F	0.0	0.0	46.3	4.4	0.0	50.7 (Dec–Feb)	PF	62°97′
**J375**	F	0.0	3.1	19.8	19.1	0.0	42.0 (Dec–Mar)	PF	63°19′
**J492**	F	0.0	13.9	24.2	10.3	0.0	48.4 (Dec–Mar)	PF	62°98′
**J503**	M	0.0	0.0	40.7	18.7	0.0	59.5 (Dec–Mar)	PF	62°84′
**Mean**		0.0	7.6±11.6	33.6±12.7	10.7±6.7	0.3±0.8	52.2±6.6		63°17′±0°75′
**1999/2000**									
**T719**	F	0.0	0.0	49.3	7.8	3.4	60.5 (Jan–May)	PF	56°46′
**T825**	F	34.6	22.8	0.0	0.0	0.0	57.4 (Feb–May)	S of SACCF-S	61°64′
**T839**	F	0.0	56.5	0.0	0.0	0.0	56.5 (Jan–Mar)	ACC to PF-S	64°57′
**T867**	F	11.3	29.6	9.9	2.2	0.0	53.0 (Jan–Apr)	ACC to PF-S	64°80′
**T875**	F	0.0	24.6	14.6	1.0	0.0	40.1 (Jan–Apr)	ACC to PF-S	59°43′
**T887**	F	0.0	36.3	18.7	0.4	0.0	55.5 (Jan–May)	ACC to PF-S	62°43′
**Mean**		7.6±13.9	28.3±18.5	15.4±18.3	1.9±3.0	0.6±1.4	53.9±6.5		61°55′±3°19′
**Overall mean**		3.8±10.2	18.0±18.3	24.5±17.8	6.3±6.7	0.4±1.1	53.1±6.3		62°36′±2°36′

The greatest proportion of seal ARS locations occurring in a particular IFZ defined their habitat group.

**Figure 2:**
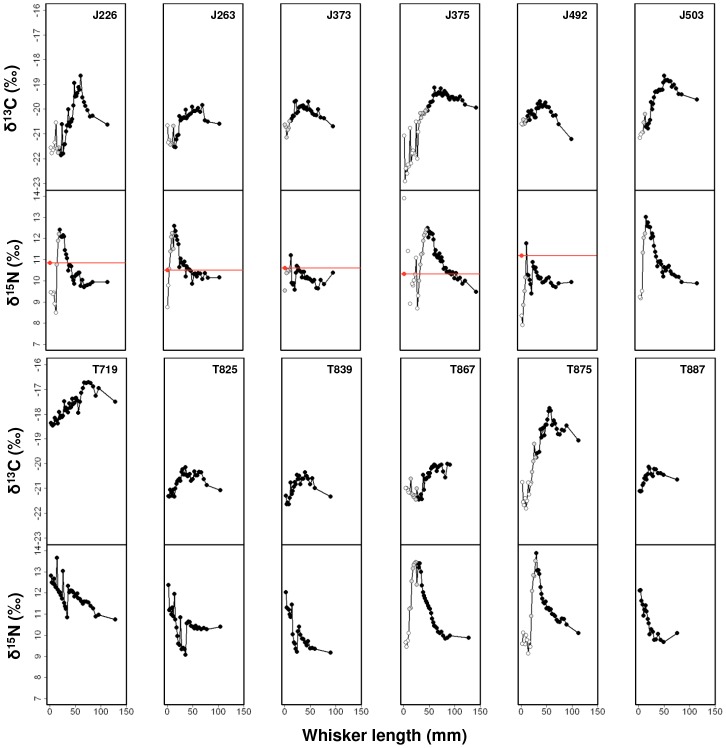
Schematic plots of δ^13^C and δ^15^N values along the post-trip whiskers of 12 sub-yearling southern elephant seals. We used stable carbon and nitrogen isotope values incorporated along the length of the whisker (mm). Values are colour-coded according to shift in food source along the temporal span of whisker presented in Fig. 1. Black symbols: *in-utero*/lactation/fasting; grey symbols: diet shift from mother’s milk and/or fasting to other food sources; open symbols: independent foraging at sea.

Isotopic values of whisker segments ranged from −22.9 to −16.6‰ (a difference of 6.3‰) for δ^13^C, and from 7.9 to 13.9 (a difference of 6‰) for δ^15^N (Fig. 2). From the distal to proximal regions of the whiskers, δ^13^C values showed an initial rise and then plateau in ^13^C abundance, before starting to fall again (Fig. 2). The number of segments and fall in ^13^C abundance varied among individuals. This pattern was even more pronounced in δ^15^N values, with seven seals showing a distinct peak and ∼4.0‰ drop in ^15^N abundance (range = 3.8 to 4.8‰). A difference of ∼4.0‰ in δ^15^N values reflects more than one trophic level of difference (∼3.0‰). The decline in δ^15^N values (grey symbols, Fig. 2) coincided with the point of interception with the pre-trip basal segment (red symbol and line, Fig. 2; n = 5), indicating a shift in food source from maternal milk and/or fasting (black symbols, Fig. 2) to independent prey acquisition (open symbols, Fig. 2).

Other seals (J373, T719, T825, T839 and T887) showed only an initial increase in ^15^N abundance along the temporal span (Fig. 2), with whiskers on average 21.4 mm shorter than that of other seals (101.2±20.9 mm, n = 5 versus 122.6±12.7 mm, n = 7; Table 3). For the latter group of seals, this indicates that the portion of whisker that contains the subsequent decline in ^15^N abundance (Fig. 1) was still beneath the skin, and thus not sampled.

**Table 3:** Stable isotopic characteristics of post-trip whiskers for each sub-yearling elephant seal (n = 12).

Seal IDNo.	Sex	Whiskerlength	Pre-trip basal segment		Post-trip whisker stable isotopic characteristics									Habitatgroup
					Overall			*In-utero*/lactation/fasting			Independent foraging at sea			
		(mm)	δ^13^C (‰)	δ^15^N (‰)	δ^13^C (‰)	δ^15^N (‰)	C:N	Length	δ^13^C (‰)	δ^15^N (‰)	Length	δ^13^C (‰)	δ^15^N (‰)	
					(range)	(range)	ratio	(mm)			(mm)			
**1995/1996**														
**J226**	F	121.0	−20.0	10.8	−20.5±1.0	10.5±1.0	2.9±0.1	95.0 (27)	−20.2±0.9	10.6±0.9	12.0 (60)	−21.4±0.5	9.1±0.4	PF
					(−21.9–−18.7)	(8.5–12.4)								
**J263**	M	112.0	−20.3	10.5	−20.6±0.5	10.9±0.8	3.0±0.1	90.0 (26)	−20.4±0.5	10.8±0.7	4.0 (2)	−21.0±0.5	9.3±0.7	ACC to PF-S
					(−21.5–−19.8)	(9.8–12.6)								
**J373**	F	105.0	−19.9	10.6	−20.2±0.4	10.2±0.4	3.0±0.1	85.0 (27)	−20.1±0.3	10.2±0.4	-	-	-	PF
					(−21.1–−19.7)	(9.6–11.2)								
**J375**	F	148.0	−19.6	10.3	−20.2±1.1	10.9±0.9	3.0±0.0	98.0 (31)	−19.5±0.2	10.9±0.8	30.0 (5)	−21.8±0.7	10.3±1.5	PF
					(−22.9–−19.1)	(8.7–12.5)								
**J492**	F	112.0	−20.4	11.2	−20.2±0.3	10.0±0.7	2.9±0.1	90.0 (24)	−20.1±0.3	10.2±0.5	4.0 (2)	−20.6±0.0	8.1±0.3	PF
					(−20.6–−19.7)	(7.9–11.8)								
**J503**	M	116.0	-	-	−19.7±0.8	11.1±1.0	3.0±0.1	102.0 (28)	−19.5±0.6	11.1±0.9	6.0 (3)	−21.1±0.1	9.3±0.2	PF
					(−21.2–−18.6)	(9.2–13.0)								
**Mean**		119.0±15.2	−20.0±0.3	10.7±0.3	−20.2±0.8	10.6±0.9		93.3±6.2	−20.0±0.6	10.7±0.8	11.2±11.0	−21.5±0.7	9.6±1.3	
					(−22.9–−18.6)	(7.9–13.0)		(27.2±2.3)			(3.6±1.8)			
**1999/2000**														
**T719**	F	132.0	-	-	−17.6±0.5	11.9±0.6	2.9±0.1	129.0 (39)*	−17.7±0.5	11.9±0.6	-	-	-	PF
					(−18.5–−16.7)	(10.8–13.7)								
**T825**	F	104.0	-	-	−20.7±0.3	10.4±0.7	2.9±0.1	104.0 (32)*	−20.7±0.4	10.4±0.7	-	-	-	S of SACCF-S
					(−21.3–−20.1)	(9.1–11.9)								
**T839**	F	89.0	-	-	−20.9±0.4	10.1±0.7	2.8±0.1	90.0 (24)*	−20.9±0.4	10.1±0.8	-	-	-	ACC to PF-S
					(−21.6–−20.4)	(9.2–11.4)								
**T867**	F	129.0	-	-	−20.7±0.5	11.4±1.3	2.9±0.1	102.0 (27)	−20.5±0.5	11.2±1.1	8.0 (4)	−21.1±0.1	9.7±0.3	ACC to PF-S
					(−21.5–−20.0)	(9.4–13.4)								
**T875**	F	120.0	-	-	−19.4±1.3	11.3±1.2	2.9±0.0	85.0 (24)	−18.6±0.5	11.5±0.9	18.0 (9)	−21.3±0.4	9.7±0.3	ACC to PF-S
					(−21.8–−17.8)	(9.1–13.9)								
**T887**	F	76.0	-	-	−20.5±0.3	10.6±0.8	2.8±0.1	76.0 (19)*	−20.6±0.3	10.7±0.8	-	-	-	ACC to PF-S
					(−21.1–−20.1)	(9.7–12.1)								
**Mean**		108.3±22.6	-	-	−19.8±1.4	11.1±1.1		93.5±12.0	−19.6±1.1	11.3±1.0	13.0±7.1	−21.4±0.4	9.7±1.0	
					(−21.8–−16.7)	(9.1–13.9)		(25.5±2.1)			(6.5±3.5)			
**Overall mean**		113.7±19.2	−20.0±0.3	10.7±0.3	−20.0±1.1	10.8±1.0		93.4±6.9	−19.9±0.8	10.8±0.9	11.7±9.5	−21.4±0.6	9.6±1.0	
					(−22.9–−16.7)	(7.9–13.9)		(26.8±2.3)			(9.1±6.4)			

* Seals showed incomplete whisker growth (mm) during *in-utero*/lactation/fasting. Results for these animals are not included in the mean and overall mean (± standard deviation**).**

The portion of post-trip whisker grown during *in-utero*/lactation/fasting ranged in length from 85 to 102 mm (mean = 93.4±6.9 mm; 77.2±5.7% of total whisker length; n = 8; Table 3) with the number of segments per whisker ranging from 24 to 31 (mean = 26.8±2.3), while the portion of post-trip whisker grown during independent foraging at sea ranged in length from 4 to 30 mm (mean = 11.7±9.5 mm; 9.1±6.4% of total whisker length; n = 7; Table 3) with the number of segments per whisker ranging from 2 to 9 (mean = 4.4±2.5).

### Foraging Habitat

The number of days that the sub-yearling elephant seals spent at sea ranged from 126 to 182 days (mean = 153.2±19.5 d; Table 1). The time spent in transit mode ranged from 12 to 76 days (mean = 50.0±15.1 d; 32.4% of all locations at sea), while the time spent in ARS mode ranged from 62 to 108 days (mean = 81.7±16.1 d; 53.2% of all locations at sea).

**Figure 3:**
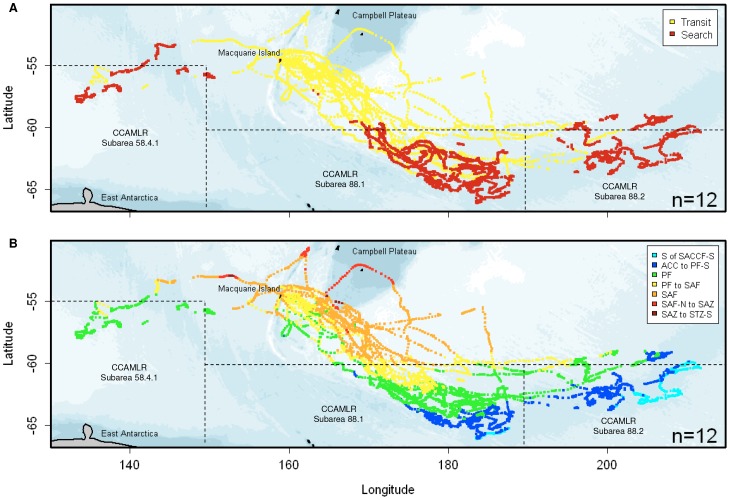
Tracks of 12 weaned southern elephant seals during their first migration from Macquarie Island. Tracks are colour-coded according to (A) behavioural state estimates from the two-state first-difference correlated random walk switching (DCRWS) model overlaid in yellow (Transit) and red (Area Restricted Search) and (B) Inter-Frontal Zones (IFZs). From south to north, IFZs included the Antarctic zone south of the southern Antarctic Circumpolar Current Front-Southern branch (S of SACCF-S), the ACC to Polar Front-Southern branch (ACC to PF-S), the PF, the PF to sub-Antarctic Front (PF to SAF), the SAF, and the SAF-Northern branch to sub-Antarctic Zone (SAF-N to SAZ). Dashed lines indicate the boundaries of CCAMLR Statistical Subareas 58.4.1, 88.1 and 88.2. All seal tracks originated and terminated at Macquarie Island, located in the South-West Pacific Ocean sector of the Southern Ocean.

Area Restricted Search locations for sub-yearlings in the 1995/1996 deployment occurred between December 1995 and April 1996, and between January and May 2000 in the 1999/2000 deployment (Table 2). Area Restricted Search locations occurred primarily at the distal portion of tracks (Fig. 3A; see Fig. S1 for individual tracks). The highest proportion of sub-yearlings (1995/1996: n = 6; 1999/2000: n = 2) utilized waters southeast of Macquarie Island, parallel with the Mid-Ocean ridge (MOR), ranging from 55°S to 66°S and 160°E to 170°W (Fig. 3A). In 1999/2000, three individuals utilized waters further to the southeast ranging from 59°S to 64°S and 160°E to 160°W, while one individual (T719) utilized waters southwest of Macquarie Island, associated with the Southeast Indian Ridge (SEIR), ranging from 53°S to 58°S and 130°E to 160°E (Fig. 3A). The majority of ARS locations of seals occurred within Commission for the Conservation of Antarctic Marine Living Resources (CCAMLR) Statistical Subareas 58.4.1, to the southwest of Macquarie Island, and 88.1 and 88.2 to the southeast (Fig. 3A).

Of the seven summarised IFZs utilised by sub-yearlings during migration from Macquarie Island (Fig. 3B), five were used by young seals while in ARS mode (Table 2). Of these, the PF was the most commonly used, with 24.5±17.8% of all ARS locations occurring in this zone, followed by the ACC to PF-S (18.0±18.3%), the PF to SAF (6.3±6.7%), the S of SACCF-S (3.8±10.2%) and lastly, the SAF (0.4±1.1%). Between deployment years, the PF was the most commonly used zone in 1995/1996 (n = 5), while the ACC to PF-S was the most commonly used zone in 1999/2000 (n = 5). A single individual in 1999/2000 however, predominantly utilized waters south of the SACCF-S, with 34.6% of all search locations occurring in this zone (Table 2). The mean latitude of ARS locations occurring in each zone ranged from 56°5′S to 63°2′S in the PF, from 59°4′S to 64°6′S in the ACC to PF-S, and 61°6′S in the S of SACCF-S. We therefore identified two main habitat groups, the ‘ACC to PF-S’ (n = 5) and the ‘PF’ (n = 6; Table 2). The small sample size of seals utilizing the region south of the SACCF-S (n = 1) however, precluded further statistical comparison of this IFZ.

### Variation in Habitat and Trophic Position (δ^13^C and δ^15^N) of Seals with the Location of Foraging

**Figure 4:**
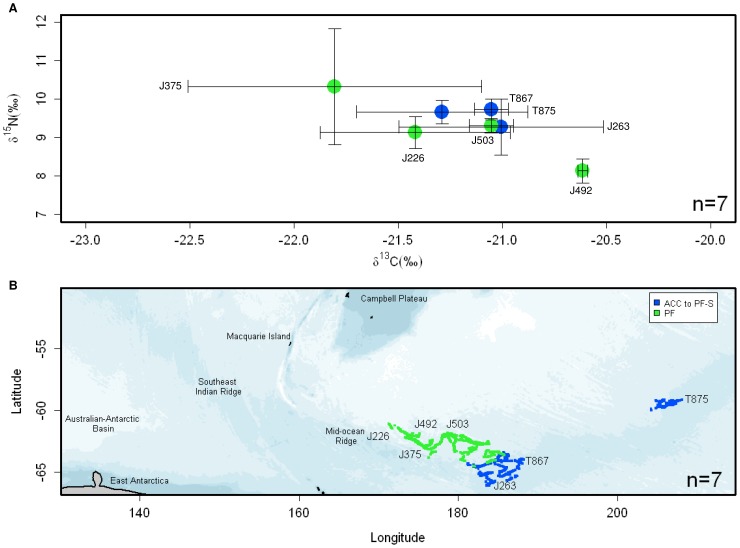
Area Restricted Search locations and whisker δ^13^C and δ^15^N values reflecting independent foraging at sea. (A) Mean δ^13^C and δ^15^N whisker values and (B) Area Restricted Search locations for 7 sub-yearling elephant seals during their first migration from Macquarie Island are colour-coded according to foraging location (Inter-Frontal Zones, IFZs, presented in Fig. 2). Bathymetric features including the Southeast Indian Ridge, Australian-Antarctic Basin and Mid-Ocean Ridge are indicated in (B). Values are mean±SD.

There was considerable overlap in δ^13^C and δ^15^N whisker values between individuals, with no significant differences in isotopic means and variances between years or IFZs detected in multivariate (MANOVA, Wilk’s λ: Year: *F*_1,2_ = 1.150, *P* = 0.426; Zone: *F*_1,2_ = 0.499, *P* = 0.650) and in uni-variate analysis (ANOVA: δ^13^C: all *P*>0.688; δ^15^N: all *P*>0.514). Mean isotopic values of sub-yearling elephant seal whiskers foraging in both the ACC to PF-S and PF were −21.2±0.4‰ (range = −21.8 to −20.6‰; a difference of 1.2‰) for δ^13^C, and 9.4±0.7‰ (range = 8.1 to 10.3‰; a difference of 2.2‰) for δ^15^N (Table 3; Fig. 4A).

As latitude is central in relating isotopic signatures in the tissues of consumers to foraging habitat in the Southern Ocean, we looked at the potential relationships between isotopic signatures (δ^13^C and δ^15^N) of elephant seal whiskers and the mean latitude of their foraging locations (*i.e.* habitat group, Table 2). No significant relationship between whisker isotopic values and mean latitude of foraging was detected (ANOVA: δ^13^C: *F*_1,5_ = 0.263, *P* = 0.630; δ^15^N: *F*_1,5_ = 0.004, *P* = 0.950), with considerable overlap in both δ^13^C and δ^15^N values and mean latitude of foraging location among seals (Fig. 4). Foraging locations of sub-yearlings encompassed a narrow latitudinal band (∼60 to 65°S), with mean latitude of locations ranging from 62°42′ to 63°19′S for seals foraging in the PF zone (n = 4), and from 59°43′ to 64°80′S for seals foraging in the ACC to PF-S zone (n = 3; Fig. 4B).

### Inferred Prey Consumption during the First 6 Months

**Figure 5:**
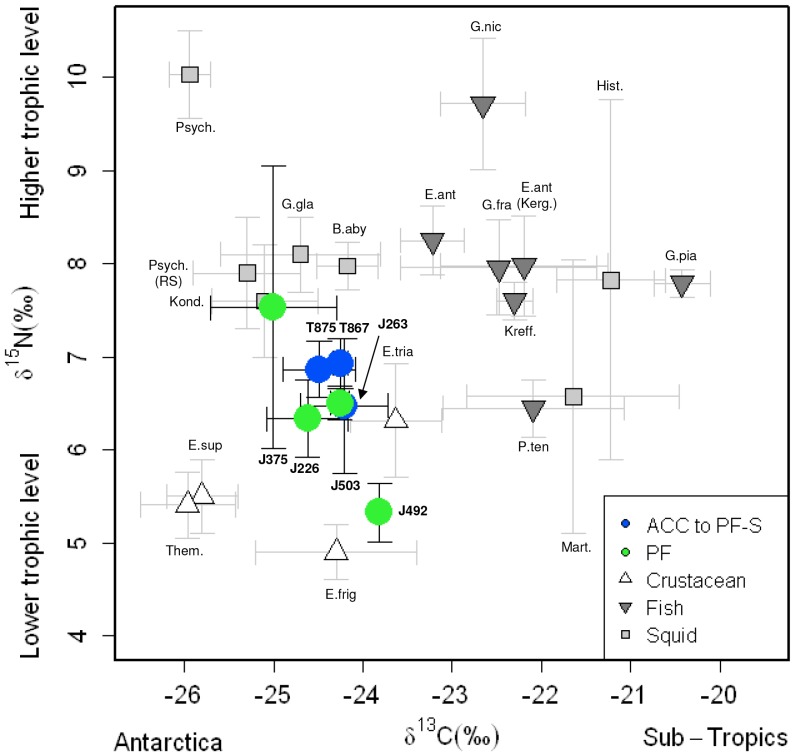
Mean δ^13^C and δ^15^N values of sub-yearling elephant seals and other Southern Ocean marine organisms. Mean δ^13^C and δ^15^N values in the whiskers of individual sub-yearlings foraging in ACC to PF-S (blue symbols) and PF (green symbols) zones, corrected for trophic discrimination by subtracting 3.2‰ and 2.8‰ from δ^13^C and δ^15^N values in Fig. 4B, respectively. Mean δ^13^C and δ^15^N values in the tissues of other Southern Ocean marine organisms ([69], [70], [71], [72], this study) were grouped into crustacean, fish and squid taxa (white, dark grey and grey symbols, respectively). E.frig: *Euphausia frigida*; E.sup: *E. superba*; E.tria: *E. triacantha*; Them: *Themisto gaudichaudii*; E.ant: *Electrona antarctica*; E.ant (Kerg.): *Electrona antarctica* (Kerguelen); G.fra: *Gymnoscopelus fraseri*; G.nic: *G. nicholsi*; G.pia: *G. piablis*; Kreff.: *Krefftichthys anderssoni*; P.ten: *Protomyctophum tenisoni*; B.aby: *Bathyteuthis abysicola*; Hist.: *Histioteuthis eltaninae*; Kond.: *Kondakovia longimana*; Psych.: *Psychroteuthis glacialis*; Psych. (RS): *Psychroteuthis glacialis* (Ross Sea); G.gla: *Galiteuthis glacialis*; Mart.: *Martialia hyadesi*. Squid beak values were corrected for the reduced ^15^N enrichment due to chitin. Values are mean±SD.

Mean δ^13^C and δ^15^N values for seal whiskers (corrected for trophic discrimination) and potential prey items were plotted together (Fig. 5; Table S1). Seals foraging in the two IFZs showed similar isotope values to a mixture of intermediate trophic level mesopelagic fish, such as *E. antarctica* (δ^13^C: −23.2‰ and δ^15^N: 8.3‰), *G. fraseri* (δ^13^C: −22.5‰ and δ^15^N value: 8.0‰), *Krefftichthys anderssoni* (δ^13^C: −22.3‰ and δ^15^N: 7.6‰), and the squids *Kondakovia longimana* (δ^13^C: −25.1‰ and δ^15^N: 7.6‰) and *Galiteuthis glacialis* (δ^13^C: −24.7‰ and δ^15^N: 8.1‰), and lower trophic level mesopelgic fish, such as *P. tensioni* (δ^13^C: −22.1‰ and δ^15^N value: 6.4‰), and the squid *M. hyadesi* (δ^13^C: −21.6‰ and δ^15^N value: 6.6‰).

The δ^13^C and δ^15^N values recorded in the seals (as shown in Fig. 5) strongly suggest that crustaceans, such as euphausiids, are consumed. This is because the isotopic values of seals are (to varying degrees) intermediate between crustaceans, such as *E. triacantha* (δ^13^C: −23.6‰ and δ^15^N value: 6.3‰), *E. superba* (δ^13^C: −25.8‰ and δ^15^N value: 5.5‰) and *T. gaudichaudii* (δ^13^C: −26.0‰ and δ^15^N value: 5.4‰), and higher trophic level fish and squid (Fig. 5). In at least four individuals (J263, J226, J503 and J492) they appear to have had some euphausiids in their diets. The interpretation of the other three individuals (J375, T867 and T875) is more ambiguous as their position on the plot could be due to either a mixture of squid, such as *P. glacialis* (δ^13^C: −25.3‰ and δ^15^N value: 7.9‰) and *M. hyadesi* or euphausiids and squid, such as *E. frigida* (δ^13^C: −24.3‰ and δ^15^N value: 4.9‰) and *P. glacialis* or even a combination of fish, squid and crustaceans.

### Variation in Habitat and Trophic Position (δ^13^C and δ^15^N) of Seals with Age

**Figure 6:**
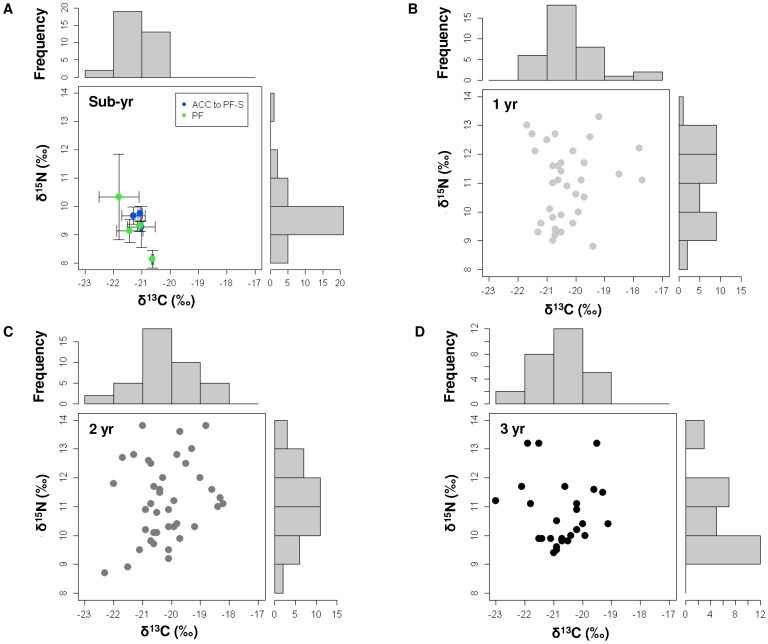
Whisker δ^13^C and δ^15^N values of juvenile southern elephant seals from Macquarie Island. Stable carbon (δ^13^C) and nitrogen (δ^15^N) isotope values from a randomly selected 2 mm section of whisker from (A) sub-yearling (n = 7; colour coded by foraging locations (IFZs) presented in Fig. 4), (B) one year (n = 35; light grey symbols), (C) two year (n = 40; dark grey symbols) and (D) three year old (n = 27; black symbols) age classes of elephant seals from Macquarie Island ([75], this study). Also shown are marginal frequency distributions for the δ^13^C and δ^15^N values for each age class.

Stable carbon values in whiskers showed significant variation with age (mixed-effects ANOVA: F_3,126_ = 10.116, *P* = 0.001), reflecting spatial variability in foraging habitat between age classes. Mean δ^13^C values ranged from −21.4±0.6‰ (range = −21.9 to −20.5‰) for sub-yearlings, compared to −20.2±0.9‰ (range = −21.7 to −17.7‰), −20.2±1.0‰ (range = −22.3 to −18.2‰) and −20.7±0.9‰ (range = −23.0 to −19.1‰) for one, two and three year olds, respectively (Fig. 6). Post-hoc analysis indicated sub-yearlings were significantly depleted in δ^13^C compared to one and two year olds (Tukey’s HSD post-hoc difference tests, both *P*<0.0001). Sub-yearlings also appeared depleted in δ^13^C compared to three olds, however no significant variation was detected (*P* = 0.06).

Stable nitrogen isotopic values in whiskers showed significant variation with age (mixed-effects ANOVA: *F*_3,126_ = 8.720, *P*<0.0001). Mean δ^15^N values ranged from 9.6±1.0‰ (range = 7.9 to 13.9.0‰) for sub-yearlings, compared to 11.0±1.3‰ (range = 8.8 to 13.3‰), 11.2±1.4‰ (range = 8.7 to 13.8‰), and 10.7±1.1‰ (range = 9.4 to 13.2‰) for one, two and three year old age classes, respectively (Fig. 6). Post-hoc analysis indicated sub-yearlings were significantly depleted in δ^15^N compared to one, two and three year old seals (Tukey’s HSD post-hoc difference tests, all *P*<0.01). Some one and two year old seals however, showed similar depleted δ^13^C and δ^15^N values as sub-yearlings, indicating overlap in both foraging habitat and trophic position.

## Discussion

The foraging range and prey intake of air-breathing marine predators is largely dictated by their physiological capabilities, most often related to body size [77], [78], [79]. The diet of young, independent offspring during their first foraging migration may therefore, differ substantially from that of older and larger conspecifics. This is particularly true for species where physiological and behavioural attributes can take several years to reach adult capacity. In southern elephant seals the dive durations and depth of newly weaned seals are known to be limited by their body size [20], [40].

Using a combination of tracking and stable isotope data, we found the diet of sub-yearling southern elephant seals from Macquarie Island, foraging predominantly in waters at or south of the PF (∼60°S) and within CCAMLR Statistical Subareas 88.1 and 88.2, to be consistent with the consumption of mesopelagic fish and squid, and crustaceans. The predominance of mesopelagic fish and squid in the diet of older juvenile and adult seals has been well documented [5], [80], [81], [82], [83], [84] however, the likely importance of crustaceans, such as euphausiids, in the diet of young seals feeding inside the CCAMLR management zone, is a significant, new finding for this species. Comparison of whisker isotopic values of sub-yearlings and older juvenile age classes (one, two and three year olds) ([75], this study) showed sub-yearlings to be relatively depleted in both δ^13^C and δ^15^N. This is indicative of younger seals (constrained by their size, dive capabilities and prey capture skills) unable to access larger, higher trophic level prey deep in the water column (>300 m), feeding closer to the surface where lower trophic level crustaceans, such as euphausiids, offer an abundant source of prey in addition to fish and squid.

Precaution must be taken in interpreting dietary trends inferred from isotopic results, as the combination of prey types using this technique can never be accurately quantified [85], [86], [87]. The most plausible explanation for the low mean δ^13^C and δ^15^N values of sub-yearlings however is the consumption of euphausiids (and copepods and amphipods). Moreover, tracking data confirms that the foraging range of sub-yearlings at this time of year overlaps with the maximum sea-ice extent and known distribution of euphausiids in this region of east Antarctica [88], [89], [90]. Further, the known dive depth and diurnal migrations of sub-yearlings (dive depth ∼100 m) [40] is consistent with the vertical distribution of krill in the Western Antarctic Peninsula during winter [91], [92].

From a conservation and management viewpoint, our findings have important implications. We provide evidence of a new crustacean (krill) predator, the southern elephant seal, within waters managed under CCAMLR. Our findings show that regions associated with the Ross Sea constitute important foraging grounds for southern elephant seals during their critical, first year of life in which they transition to independent foraging.

### Whisker Growth Dynamics

Bio-logging and natural biochemical tracers are increasingly being used to provide spatially explicit dietary information for highly migratory marine predators [93], [94], [95], [96]. Whiskers, collected from animals tracked by satellite, contain a time-series of stable isotope ratios of carbon and nitrogen which can potentially be related to individual telemetry tracks, establishing a means to link diet to remote feeding grounds. To interpret this dietary information in a spatio-temporal context however requires knowledge of the growth history of the whisker [36], [97], [98].

Accounting for the growth dynamics of whiskers enables the correct interpretation of the time-series of diet information incorporated along the length of the whisker [36]. In all sub-yearling elephant seals we found a similar pattern in isotopic enrichment along the temporal span of the whisker, *i.e.* from the distal to proximal region. However, this pattern was more pronounced in δ^15^N values, with an initial rise and distinct peak in ^15^N abundance indicative of the shift in food source from *in-utero* development to mother’s milk during lactation. Nursing offspring essentially feed at a higher trophic level than their mothers do as has been shown in several species, including pinnipeds [99], [100], [101]. In northern elephant seals, Habran et al. [100] found young pups to be increasingly enriched in ^15^N compared to that of their mothers from early (+0.6‰, day 5) to late lactation (+1.3‰, day 22), while in Steller sea lions (*Eumetopias jubatus*), Stegall et al. [101] recorded the root of the whisker (representing current growth) of young pups to be ^15^N enriched (+2.0‰) over their diet (ingested mother’s milk) during lactation.

Of the twelve sub-yearlings with concurrent isotopic and tracking data, seven seals showed a subsequent fall in δ^15^N values, equivalent to more than one trophic level of a difference (∼3‰) and indicative of a diet shift from mother’s milk and/or fasting to free ranging prey. We suggest that the reason we do not see this decline in δ^15^N values in the other five seals is that slower whisker growth meant that the new material, synthesized after weaning, never appeared above the skin and was therefore not sampled. The portion of whisker grown during independent foraging at sea accounted for 11.7±9.5 mm or 9.1±6.4% of total growth, indicating that an average of 12 mm of growth is contained under the skin for the latter group of seals. In contrast, the portion of post-trip whisker grown during *in-utero*/lactation/fasting accounted for 93.4±6.9 mm or 77.2±5.7% of total whisker growth (n = 8). In elephant seals, whiskers are established early during *in-utero* development with foetal whiskers growing as much as 27 mm in length, and are not shed during their annual pelage moult but randomly after seals are at least two years of age [102]. Moreover, whisker growth rates (0.87 mm per day) of new born, nursing bearded seals (*Erignathus barbatus*) suggest periods of rapid, somatic growth may be reflected in the growth of the whiskers [103]. These results therefore suggest that like other phocid species, the rapid accumulation of energy reserves as blubber by nursing elephant seals [11], [104], may be reflected in the growth of the whiskers.

In summary, we found whiskers to be extremely useful tools for accessing time integrated diet information of elephant seals during their first year of life as we can trace the origin of the signature incorporated along the length of the whisker, *i.e.* from maternal investment to independent foraging. A significant finding of this study was that whiskers collected after the first foraging trip were predominantly still composed of *in-utero* and pre-weaning material as a result of relatively slow whisker growth rates during the 3 to 4 month foraging trip. The results of this study also highlight the need to sample plucked and not cut whiskers for future isotopic dietary analysis in order to capture the most recent isotopic information contained in the root of the whisker under the skin.

### Foraging Habitat

The foraging behaviour of a predator can change in response to the distribution of prey resources in a large heterogeneous environment. Identifying changes in the movement behaviour of a predator can therefore be informative as to the distribution and consumption of prey resources at a range of temporal scales. Over a period of 4 to 6 months satellite tracking data showed that sub-yearling elephant seals dispersed, in some cases thousands of kilometres, to the southeast of Macquarie Island ([10], [40], this study) apart from one individual which travelled to the southwest of Macquarie Island. Area Restricted Search locations, used as a proxy for foraging areas of sub-yearling elephant seals, occurred at the distal portion of tracks.

Foraging locations in the southeast group were primarily associated with ACC frontal branches of the PF (PF-S to PF-N, summarised as the PF; n = 5) and the SACCF (SACCF-S to PF-S, summarised as the ACC to PF-S; n = 6) and bathymetric features such as the MOR. The foraging locations of the single seal which travelled to the southwest of Macquarie Island were primarily associated with frontal branches of the PF and the SEIR. In this study, the foraging locations of only one seal were primarily associated with waters to the south of the sBdy (south of sBdy to SACCF-S, summarised as S of SACCF-S).

The movement and foraging behaviour of sub-yearling elephant seals from this site has been previously investigated [10], [21], [40], [105]. Two studies in particular, detail the movement, foraging areas [10], [40] and dive behaviour [40] of seals, including animals from this study. In this study, we separated travel into two phases, transit and ARS. Area Restricted Search behaviour is thought to occur in response to the patchy distribution of resources in an environment [106]. This behaviour corresponds with periods of reduced travel speed and increased turning rate, which are more likely to be associated with movements associated with feeding as opposed to transit [48]. McConnell et al. [10] and Hindell et al. [40] separated travel into three phases (initial outbound transit, intermediate movement and final return transit) based upon daily travel rates for a large number of sub-yearlings (n = 30 for both studies). Intermediate tracks, presumed to represent feeding, corresponded to slower and less directed travel, interrupted by occasional bouts of increased travel [10], [40]. The dive behaviour of seals during this time consisted of relatively shallow and short dives (117±48 m and 5.9±1.4 min, respectively; [40]). Concentrated activity (locations of feeding) were centred on localised patches up to 1900 km from Macquarie Island, with the southern boundary of tracks in the southeast group aligned with the SACCF [10], [40]. These foraging areas (mean duration 67 days) matched well with the ARS areas (mean duration 81 days) of the sub-yearlings in this study.

### Variation in Habitat (δ^13^C) of Seals with the Location of Foraging

We found that the most depleted δ^13^C values incorporated along the temporal span of the whisker (as represented by the growth of the whisker) were contained in the portion of whisker grown during independent foraging at sea (−21.4±0.6‰, n = 7; Fig. 2), while the most enriched δ^13^C values were contained in the portion of whisker grown during *in-utero*/lactation/fasting (−19.8±1.1‰, n = 12; Table 2). In oceanic waters of the Southern Ocean, the POM δ^13^C values become more depleted with increasing latitudes, and these latitudinal changes are subsequently transferred to higher levels within the food chain [67], [68]. The decline in δ^13^C values towards the proximal region of the whisker is therefore consistent with the southward migration of seals (outward transit tracks) to high latitude foraging areas (ARS tracks) located at or south of the PF (∼60°S) to the southeast of Macquarie Island (Fig. 4B; Fig. S1.).

It remains unclear to what degree young seals feed on the outward transit leg of their foraging trips [19] however it is most likely that sub-yearlings are in a state of transition from mother’s milk and/or fasting to free ranging prey, as indicated by the concurrent ∼3‰ drop in δ^15^N and reduced whisker growth (∼10 mm), at this time (Fig. 3). However, δ^13^C values related to independent foraging appear to stabilise at ∼−21.0‰, presumably after seals have reached their main foraging grounds. We are therefore fairly certain the δ^13^C values contained in the portion of whisker grown during independent foraging at sea are representative of core foraging habitat use, namely waters of the PF and ACC to PF-S.

There was considerable overlap in the δ^13^C signatures of sub-yearlings related to independent foraging, both between years and foraging locations (IFZs: PF = −21.5±0.5‰, n = 4; ACC to PF-S = −21.2±0.4‰, n = 3), indicating that even though seals were using similar latitudes (62°89′±1°77′; n = 7), they were in fact in different water masses, and there was no difference in δ^13^C between those water masses. The structure and flow of the ACC is complex, consisting of multiple frontal filaments or branches that are strongly influenced by bathymetry [52]. The PF marks the southern boundary of the PF to SAF zone and the beginning of the ACC to PF-S zone, while the sBdy delimits the southern boundary of the ACC to PF-S and the beginning of the S of SACCF-S zone. In the southwest Pacific sector, the frontal branches of the PF and SACCF merge to form a single frontal zone on the northern slope of the MOR near 170°E or where diverted to the south by obstacles like the Campbell Plateau. Frontal branches however, are clearly separated over deep ocean basins, such as the Australian – Antarctic Basin to the southwest of Macquarie Island [52]. As a consequence, the magnitude of the latitudinal variation in the boundaries of the PF and SACCF is much greater to the southwest of Macquarie Island than to the southeast of Macquarie Island [50], [51], [52], [53], [107]. This indicates that δ^13^C signatures are not adequate to resolve habitat (water mass) at this scale and consequently, seals located to the southeast of Macquarie Island and predominantly associated with frontal branches of the PF and SACCF (*i.e.* PF and ACC to PF-S zones, respectively) and the MOR show similar δ^13^C values.

### Inferred Prey Consumption during the First Six Months

Very little is known about the diet of southern elephant seals during the course of their migrations, particularly in relation to core foraging areas. Lavaged stomachs of both juvenile and adult seals returning to colonies, which represent the most recent prey intake at the end of foraging trips, consist largely of mesopelagic fish and squid at Macquarie Island [5], [22], [80], [108], and other populations across the Southern Ocean [23], [81], [83], [84], [109], [110], while an increasing amount of inferential data from biochemical tracers, such as stable isotopes and fatty acids augment the dietary trends identified by stomach content analysis [15], [70], [75], [111]. There is however, very little information available on the diet of elephant seals during their first foraging migration [81]. The whisker isotopic signatures of sub-yearlings provided dietary information corresponding to at least the first half of their foraging trips. Stable nitrogen values spanned more than one trophic level, with considerable overlap in δ^15^N values of seals both between years and foraging locations (IFZs) suggesting that all seals fed at a similar trophic level irrespective of foraging habitat. However, there was considerable individual variability in their diet. Pronounced individual variability has also been described in diving behaviour within water masses for this species [24]. Individual specialisation in diet increases the niche breadth for a population and may offer some buffering against a changing resources base [103].

The trophic position of seals, which foraged to the southeast of Macquarie Island in waters at or south of the PF and within CCAMLR Subareas 88.1 and 88.2 (>60°S; Fig. 3) were consistent with the consumption of a mixture of intermediate trophic level mesopelagic fish and squid (δ^15^N: ∼8‰, such as *E. antarctica, G. fraseri*, *K. anderssoni, G. glacialis*, *K. longimana* and *P. glacialis*), lower trophic level mesopelagic fish and squid (δ^15^N: ∼5–7‰, such as *M. hyadesi* and *P. tenisoni*) and lower trophic level crustaceans (δ^15^N: <6‰, such as euphausiids, copepods and amphipods) characteristic of that sampled within colder, high latitude eastern Antarctic waters [88]. The consumption of mesopelagic fish and squid is consistent with the dietary trends of elephant seals determined in previous studies. However, sub-yearlings showed lower mean δ^15^N signatures compared to older juvenile (Fig. 6) [75] and adult seals [70]. When we compare the isotopic signatures of sub-yearlings and potential prey, the most parsimonious explanation is the consumption of lower trophic level crustaceans, such as euphausiids, in addition to fish and squid. In addition, stable isotope data provided dietary information relating to the first half of foraging trips, with the most recent isotopic values contained in the root of the whisker under the skin. Therefore, this may only be a conservative estimate of the level of crustacean consumption by seals in this study and requires further investigation.

Caution must be taken however, in over-interpretation of these observed dietary trends as prey data (other than squid beak data for *H. eltaninae* and *M. hyadesi*) are taken from outside the foraging range of juvenile elephant seals from this site [14], [21] and therefore, requires further examination. Moreover, isotopic fractionation values for keratinous tissues vary among species and studies [112] thus, factors determined for captive pinnipeds (as applied in this study) may not accurately represent wild populations. Nevertheless, the presence of myctophid fish, such as *Electrona* and *Gymnoscopelus* spp. [5], [81] and of squid, such as *P. glacialis* and *H. eltaninae* in the stomach contents of older juveniles from Macquarie Island [22] and Heard Island [81], confirms the consumption of mesopelagic prey by sub-yearlings. Moreover, similarities in foraging behaviour [40] and trophic level among seals and one of the most specialised consumers of myctophids and squid, the king penguin (*Aptenodytes patagonicus*) [113], [114], [115], which forages in similar regions of the PF south of Macquarie Island [116], also provides evidence that sub-yearlings fed at depths where similar mesopelagic fish and squid prey were accessible.

Crustaceans have been reported in the diet of elephant seals [5], [23], [80], [81], however, this has usually been attributed to incidental or secondary consumption. Nevertheless, stomach content analysis revealed higher proportions of crustaceans in the diet of one and two year-old seals compared to that of three year-olds [5], and were reported as primary prey of elephant seals by Green and Burton [80]. *Euphausia triacantha* generally occurs in waters between 50°S and 65°S, with vertical distribution between 250 to 750 m during the day and above 250 m at night [117]. *Euphausia superba* is predominantly herbivorous [118], [119], while more northern species, such as *E. triacantha*, are carnivorous [120], which explains its higher trophic position relative to *E. superba*. Due to the diurnal changes in the abundance and distribution of euphausiids [121], [122], sub-yearlings, which are smaller and cannot dive as deep or for as long as larger seals [20], [40], may encounter euphuasiid species at densities sufficiently large at shallower depths to make these important prey items.

Moreover, the southern boundary of foraging areas of sub-yearlings aligned with the sBdy (∼65°S; Fig. 2), coinciding with the maximum sea-ice extent [90] and known distribution of Antarctic krill (*E. superba*) in east Antarctic waters during late summer/early winter [88], [89]. Collectively, these results indicate that lower trophic level crustaceans, namely euphausiids, may form an important part of the diet for young seals. Consequently, sub-yearlings may be an important krill predator that should be taken into account within the CCAMLR management zone. These areas are important to elephant seals during the transition to independent foraging, and other marine predators of Macquarie Island, which exploit similar areas to the south of Macquarie Island (*e.g.* king penguins and royal penguins, *Eudyptes schlegeli*) [123], [124].

### Variation in Habitat and Trophic Position (δ^13^C and δ^15^N) of Seals with Age

Ontogenetic changes in movement patterns, foraging habitat use and diet have been reported for older juvenile southern elephant seals from Macquarie Island [5], [18], [21], [22], [75], other populations [80], [81] and in their northern counterpart, the northern elephant seal [125]. Sub-yearlings showed more depleted δ^13^C and δ^15^N values than older juvenile seals, indicating ontogenetic segregation in both foraging range and trophic position, respectively. These observed isotopic differences are in good agreement with sub-yearlings feeding at a lower trophic level than older seals, since their size, dive capabilities and predation skills are limited [20], [40]. The increased diving capabilities of elephant seals with increasing age are well documented [126], and may give older juveniles and adults a substantial advantage in capturing prey found at greater depths. Indeed, higher trophic level cephalopod prey of elephant seals, such as *P. glacialis* and *Alluroteuthis antarcticus* [22], [80], occur at high densities deep in the water column (500 to 1000 m). Sub-yearling elephant seals, limited to some extent by their physiological capabilities, are restricted to the upper 300 m of the water column (mostly 100 to 200 m depth) [40]. Lower trophic level pelagic prey, such as smaller-sized myctophid fish and crustaceans, which occur in high densities in the upper limits of the water column, may therefore provide an abundant and easily accessible source of prey for smaller seals. Crustaceans may, therefore, form a significant part of the diet of some sub-yearlings.

## Supporting Information

Figure S1 **Tracks overlaid with state estimates from the two-state first-difference correlated random walk switching (DCRWS) model.** Tracks of 12 weaned southern elephant seals during their first foraging migration from Macquarie Island, colour coded by state estimates. Grey: transit locations; light blue, blue and green: Area Restricted Search (ARS) locations for S of SACCF-S, ACC to PF-S and PF zones, respectively.
(PDF)

Table S1 **Stable carbon (δ^13^C) and nitrogen (δ^15^N) values of various marine organisms from the Southern Ocean.**
(PDF)

## References

[1] Constable AJ, Nicol S, Strutton PG (2003) Southern Ocean productivity in relation to spatial and temporal variation in the physical environment. Journal of Geophysical Research: Oceans 108.

[2] Bost CA, Cotté C, Bailleul F, Cherel Y, Charrassin JB, et al (2009) The importance of oceanographic fronts to marine birds and mammals of the southern oceans. Journal of Marine Systems 78: 363–376.

[3] Reid K, Croxall JP (2001) Environmental response of upper trophic-level predators reveals a system change in an Antarctic marine ecosystem. Proceedings: Biological Sciences 268: 377–384. 11270434 10.1098/rspb.2000.1371 PMC1088617

[4] Iverson SJ, Arnould JPY, Boyd IL (1997) Milk fatty acid signatures indicate both major and minor shifts in the diet of lactating Antarctic fur seals. Canadian Journal of Zoology 75: 188–197.

[5] Field IC, Bradshaw CJA, van den Hoff J, Burton HR, Hindell MA (2007) Age-related shifts in the diet composition of southern elephant seals expand overall foraging niche. Marine Biology 150: 1441–1452.

[6] Lindström J (1999) Early development and fitness in birds and mammals. Trends in Ecology & Evolution 14: 343–348. 10441307 10.1016/s0169-5347(99)01639-0

[7] Alerstam T, Hedenström A, Åkesson S (2003) Long-distance migration: evolution and determinants. Oikos 103: 247–260.

[8] Paradis E, Baillie SR, Sutherland WJ, Gregory RD (1998) Patterns of natal and breeding dispersal in birds. Journal of Animal Ecology 67: 518–536.

[9] Breed G, Bowen W, Leonard M (2011) Development of foraging strategies with age in a long-lived marine predator. Marine Ecology Progress Series 431: 267–279.

[10] McConnell B, Fedak M, Burton HR, Engelhard GH, Reijnders PJH (2002) Movements and foraging areas of naive, recently weaned southern elephant seal pups. Journal of Animal Ecology 71: 65–78.

[11] Arnbom T, Fedak MA, Boyd IL, McConnell BJ (1993) Variation in weaning mass of pups in relation to maternal mass, postweaning fast duration, and weaned pup behaviour in southern elephant seals (*Mirounga leonina*) at South Georgia. Canadian Journal of Zoology 71: 1772–1781.

[12] Arnbom T, Fedak MA, Boyd IL (1997) Factors affecting maternal expenditure in southern elephant seals during lactation. Ecology 78: 471–483.

[13] McMahon CR, Bester MN, Burton HR, Hindell MA, Bradshaw CJA (2005) Population status, trends and a re-examination of the hypotheses explaining the recent declines of the southern elephant seal *Mirounga leonina* . Mammal Review 35: 82–100.

[14] Field IC, Bradshaw CJA, Burton HR, Sumner MD, Hindell MA (2005) Resource partitioning through oceanic segregation of foraging juvenile southern elephant seals (*Mirounga leonina*). Oecologia 142: 127–135. 15365810 10.1007/s00442-004-1704-2

[15] Bradshaw CJA, Hindell MA, Best NJ, Phillips KL, Wilson G, et al (2003) You are what you eat: describing the foraging ecology of southern elephant seals (*Mirounga leonina*) using blubber fatty acids. Proceedings of the Royal Society of London Series B-Biological Sciences 270: 1283–1292. 10.1098/rspb.2003.2371 PMC1691367 12816642

[16] Bradshaw CJA, Hindell MA, Sumner MD, Michael KJ (2004) Loyalty pays: potential life history consequences of fidelity to marine foraging regions by southern elephant seals. Animal Behaviour 68: 1349–1360.

[17] Robinson PW, Costa DP, Crocker DE, Gallo-Reynoso JP, Champagne CD, et al (2012) Foraging behavior and success of a mesopelagic predator in the northeast Pacific Ocean: Insights from a data-rich species, the northern elephant seal. PLoS ONE 7: e36728. 22615801 10.1371/journal.pone.0036728 PMC3352920

[18] Field IC, Bradshaw CJA, Burton HR, Hindell MA (2007) Differential resource allocation strategies in juvenile elephant seals in the highly seasonal Southern Ocean. Marine Ecology Progress Series 331: 281–290.

[19] Thums M, Bradshaw CJA, Hindell MA (2011) In situ measures of foraging success and prey encounter reveal marine habitat-dependent search strategies. Ecology 92: 1258–1270. 21797154 10.1890/09-1299.1

[20] Irvine LG, Hindell MA, van den Hoff J, Burton HR (2000) The influence of body size on dive duration of underyearling southern elephant seals (*Mirounga leonina*). Journal of Zoology 251: 463–471.

[21] van den Hoff J, Burton HR, Hindell MA, Sumner MD, McMahon CR (2002) Migrations and foraging of juvenile southern elephant seals from Macquarie Island within CCAMLR managed areas. Antarctic Science 14: 134–145.

[22] van den Hoff J (2004) A comparative study of the cephalopod prey of Patagonian toothfish (*Dissostichus eleginoides*) and southern elephant seals (*Mirounga leonina*) near Macquarie Island. Polar Biology 27: 604–612.

[23] van den Hoff J, Burton H, Davies R (2003) Diet of male southern elephant seals (*Mirounga leonina* L.) hauled out at Vincennes Bay, East Antarctica. Polar Biology 26: 27–31.

[24] Field I, Hindell M, Slip D, Michael K (2001) Foraging strategies of southern elephant seals (*Mirounga leonina*) in relation to frontal zones and water masses. Antarctic Science 13: 371–379.

[25] Best PB, Schell DM (1996) Stable isotopes in southern right whale (*Eubalaena australis*) baleen as indicators of seasonal movements, feeding and growth. Marine Biology 124: 483–494.

[26] Rubenstein DR, Hobson KA (2004) From birds to butterflies: animal movement patterns and stable isotopes. Trends in Ecology and Evolution 19: 256–263. 16701265 10.1016/j.tree.2004.03.017

[27] Bearhop S, Thompson DR, Waldron S, Russell IC, Alexander G, et al (1999) Stable isotopes indicate the extent of freshwater feeding by cormorants *Phalacrocorax carbo* shot at inland fisheries in England. Journal of Applied Ecology 36: 75–84.

[28] Hobson KA (1999) Tracing origins and migration of wildlife using stable isotopes: a review. Oecologia 120: 314–326. 28308009 10.1007/s004420050865

[29] Koch PL, Heisinger J, Moss C, Carlson RW, Fogel ML, et al (1995) Isotopic tracking of change in diet and habitat use in African elephants. Science 267: 1340–1343. 17812610 10.1126/science.267.5202.1340

[30] Hobson KA, Schell DM, Renouf D, Noseworthy E (1996) Stable carbon and nitrogen isotopic fractionation between diet and tissues of captive seals: implications for dietary reconstructions involving marine mammals. Canadian Journal of Fisheries and Aquatic Sciences 53: 528–533.

[31] Fry B, Sherr EB (1984) δ^13^C measurements as indicators of carbon flow in marine and freshwater ecosystems. Contributions in Marine Science 27: 13–47.

[32] Peterson BJ, Fry B (1987) Stable Isotopes in Ecosystem Studies. Annual Review of Ecology and Systematics 18: 293–320.

[33] Minagawa M, Wada E (1984) Stepwise enrichment of δ^15^N along food chains: Further evidence and the relation between δ^15^N and animal age. Geochimica et Cosmochimica Acta 48: 1135–1140.

[34] McCutchan JH, Lewis WM, Kendall C, McGrath CC (2003) Variation in trophic shift for stable isotope ratios of carbon, nitrogen, and sulfur. Oikos 102: 378–390.

[35] Hobson KA, Welch, H E. (1992) Determination of trophic relationships within a high Arctic marine food web using δ^13^C and δ^15^N analysis Marine Ecology Progress Series 84 9–18.

[36] Hirons A, C., Schell D, M., St Aubin D (2001) J (2001) Growth rates of vibrissae of harbor seals (*Phoca vitulina*) and Steller sea lions (*Eumetopias jubatus*). Canadian Journal of Zoology 79: 1053.

[37] Fedak MA, Lovell P, McConnell BJ (1996) MAMVIS: A Marine Mammal Behaviour Visualization System. The Journal of Visualization and Computer Animation 7: 141–147.

[38] Baker JR, Fedak MA, Anderson SS, Arnbom T, Baker R (1990) Use of a tiletamine-zolazepam mixture to immobolize wild gray seals and southern elephants seals. Veterinary Record 126: 75–77. 2309387

[39] Fedak MA, Anderson SS, Curry MG (1983) Attachment of a radio tag to the fur of seals. Journal of Zoology 200: 298–300.

[40] Hindell MA, McConnell BJ, Fedak MA, Slip DJ, Burton HR, et al (1999) Environmental and physiological determinants of successful foraging by naive southern elephant seal pups during their first trip to sea. Canadian Journal of Zoology 77: 1807–1821.

[41] Lea M-A, Nichols PD, Wilson G (2002) Fatty acid composition of lipid-rich myctophids and mackerel icefish (*Champsocephalus gunnari*) - Southern ocean food-web implications. Polar Biology 25: 843–854.

[42] Duhamel G, Koubbi P, Ravier C (2000) Day and night mesopelagic fish assemblages off the Kerguelen Islands (Southern Ocean). Polar Biology 23: 106–112.

[43] Field IC, Bradshaw CJA, McMahon CR, Harrington J, Burton HR (2002) Effects of age, size and condition of elephant seals (*Mirounga leonina*) on their intravenous anaesthesia with tiletamine and zolazepam. Veterinary Record 151: 235–240. 12219901 10.1136/vr.151.8.235

[44] McMahon CR, Burton H, McLean S, Slip D, Bester M (2000) Field immobilisation of southern elephant seals with intravenous tiletamine and zolazepam. Veterinary Record 146: 251–254. 10737295 10.1136/vr.146.9.251

[45] Hosie G, Koubbi P, Riddle M, Ozouf-Costaz C, Moteki M, et al (2011) CEAMARC, the Collaborative East Antarctic Marine Census for the Census of Antarctic Marine Life (IPY # 53): An overview. Polar Science 5: 75–87.

[46] Moteki M, Koubbi P, Pruvost P, Tavernier E, Hulley P-A (2011) Spatial distribution of pelagic fish off Adélie and George V Land, East Antarctica in the austral summer 2008. Polar Science 5: 211–224.

[47] Jonsen ID, Flemming JM, Myers RA (2005) Robust state-space modeling of animal movement data. Ecology 86: 2874–2880.

[48] Morales JM, Haydon DT, Frair J, Holsinger KE, Fryxell JM (2004) Extracting more out of relocation data: building movement models as mixtures of random walks. Ecology 85: 2436–2445.

[49] Jonsen ID, Basson M, Bestley S, Bravington MV, Patterson TA, et al (2013) State-space models for bio-loggers: A methodological road map. Deep-Sea Research Part II: Topical Studies in Oceanography 88–89: 34–46.

[50] Sokolov S, Rintoul SR (2009) Circumpolar structure and distribution of the Antarctic Circumpolar Current fronts: 2. Variability and relationship to sea surface height. Journal of Geophysical Research 114: C11019.

[51] Sokolov S, Rintoul SR (2009) Circumpolar structure and distribution of the Antarctic Circumpolar Current fronts: 1. Mean circumpolar paths. Journal of Geophysical Research 114: C11018.

[52] Sokolov S, Rintoul SR (2007) Multiple Jets of the Antarctic Circumpolar Current South of Australia. Journal of Physical Oceanography 37: 1394–1412.

[53] Sokolov S, Rintoul SR (2007) On the relationship between fronts of the Antarctic Circumpolar Current and surface chlorophyll concentrations in the Southern Ocean. Journal of Geophysical Research 112: C07030.

[54] Kojadinovic J, Richard P, Le Corre M, Cosson RP (2008) Effects of lipid extraction on δ^13^C and δ^15^N values in seabird muscle, liver and feathers. Waterbirds: The International Journal of Waterbird Biology 31: 169–178.

[55] Hobson KA, Cherel Y (2006) Isotopic reconstruction of marine food webs using cephalopod beaks: new insight from captively raised *Sepia officinalis* . Canadian Journal of Zoology 84: 766–770.

[56] Cherel Y, Fontaine C, Jackson GD, Jackson CH, Richard P (2009) Tissue, ontogenic and sex-related differences in δ^13^C and δ^15^N values of the oceanic squid *Todarodes filippovae* (Cephalopoda: Ommastrephidae). Marine Biology 156: 699–708.

[57] Miserez A, Li Y, Waite JH, Zok F (2007) Jumbo squid beaks: inspiration for design of robust organic composites. Acta Biomaterialia 3: 139–149. 17113369 10.1016/j.actbio.2006.09.004

[58] McMahon CR, Bradshaw CJA (2004) Harem choice and breeding experience of female southern elephant seals influence offspring survival. Behavioral Ecology and Sociobiology 55: 349–362.

[59] Hirons A (2001) Trophic dynamics of pinniped populations in Alaskan waters using stable carbon and nitrogen isotope ratios [PhD]. Fairbanks: University of Alaska.

[60] Hobson KA, Alisauskas RT, Clark RG (1993) Stable-nitrogen isotope enrichment in avian tissues due to fasting and nutritional stress: implications for isotopic analyses of diet. The Condor 95: 388–394.

[61] Stegall VK, Farley SD, Rea LD, Pitcher KW, Rye RO, et al (2008) Discrimination of carbon and nitrogen isotopes from milk to serum and vibrissae in Alaska Steller sea lions (*Eumetopias jubatus*). Canadian Journal of Zoology-Revue Canadienne De Zoologie 86: 17–23.

[62] Rau GH, Sweeney RE, Kaplan IR (1982) Plankton ^13^C : ^12^C ratio changes with latitude: differences between northern and southern oceans. Deep Sea Research 29: 1035–1039.

[63] Rau GH, Takahashi T, Marais DJD (1989) Latitudinal variations in plankton δ^13^C: implications for CO_2_ and productivity in past oceans. Nature 341: 516–518. 11536614 10.1038/341516a0

[64] Goericke R, Fry B (1994) Variations of marine plankton δ^13^C with latitude, temperature, and dissolved CO_2_ in the world ocean Global Biogeochemical Cycles. 8: 85–90.

[65] Popp BN, Trull T, Kenig F, Wakeham SG, Rust TM, et al (1999) Controls on the carbon isotopic composition of Southern Ocean phytoplankton. Global Biogeochem Cycles 13: 827–843.

[66] Trull TW, Armand L (2001) Insights into Southern Ocean carbon export from the δ^13^C of particles and dissolved inorganic carbon during the SOIREE iron release experiment. Deep-Sea Research Part II: Topical Studies in Oceanography 48: 2655–2680.

[67] Cherel Y, Phillips RA, Hobson KA, McGill R (2006) Stable isotope evidence of diverse species-specific and individual wintering strategies in seabirds. Biology Letters 2: 301–303. 17148388 10.1098/rsbl.2006.0445 PMC1618904

[68] Cherel Y, Hobson K (2007) A (2007) Geographical variation in carbon stable isotope signatures of marine predators: a tool to investigate their foraging areas in the Southern Ocean. Marine Ecology Progress Series 329: 281–287.

[69] Schmidt K, McClelland JW, Mente E, Montoya JP, Atkinson A, et al (2004) Trophic-level interpretation based on δ^15^N values: implications of tissue-specific fractionation and amino acid composition. Marine Ecology Progress Series 266: 43–58.

[70] Cherel Y, Ducatez S, Fontaine C, Richard P, Guinet C (2008) Stable isotopes reveal the trophic position and mesopelagic fish diet of female southern elephant seals breeding on the Kerguelen Islands. Marine Ecology Progress Series 370: 239–247.

[71] Cherel Y (2008) Isotopic niches of emperor and Adelie penguins in Adelie Land, Antarctica. Marine Biology 154: 813–821.

[72] Bury SJ, Pinkerton MH, Thompson DR, Hanchet S, Brown J, et al. (2008) Trophic study of Ross Sea Antarctic toothfish (*Dissostichus mawsoni*) using carbon and nitrogen stable isotopes. Document WG-EMM-08/27 CCAMLR, Hobart, Australia.

[73] Rodhouse PG (1989) Antarctic cephalopods: a living marine resource? Ambio 18: 56–59.

[74] Clarke MR, editor (1986) A handbook for the identification of cephalopods beaks. Oxford: Clarendon.

[75] Newland C, Field I, Cherel Y, Guinet C, Bradshaw C, et al (2011) Diet of juvenile southern elephant seals reappraised by stable isotopes in whiskers. Marine Ecology Progress Series 424: 247–258.

[76] R Development Core Team (2012) R: A Language and Environment for Statistical Computing.

[77] Schreer JF, Kovacs KM (1997) Allometry of diving capacity in air-breathing vertebrates. Canadian Journal of Zoology 75: 339–358.

[78] Burns JM (1999) The development of diving behavior in juvenile Weddell seals: Pushing physiological limits in order to survive. Canadian Journal of Zoology 77: 737–747.

[79] Weise MJ, Harvey JT, Costa DP (2010) The role of body size in individual-based foraging strategies of a top marine predator. Ecology 91: 1004–1015. 20462115 10.1890/08-1554.1

[80] Green K, Burton HR (1993) Comparison of the stomach contents of southern elephant seals, *Mirounga leonina*, at Macquarie and Heard Islands. Marine Mammal Science 9: 10–22.

[81] Slip D (1995) The diet of southern elephant seals (*Mirounga leonina*) from Heard Island Canadian Journal of Zoology. 73: 1519–1528.

[82] Daneri GA, Coria NR (1992) The diet of Antarctic fur seals, *Arctocephalus gazella*, during the summer-autumn period at Mossman Peninsula, Laurie Island (South Orkneys). Polar Biology 11: 565–566.

[83] Daneri G, Carlini A, Rodhouse PG (2000) Cephalopod diet of the southern elephant seal, *Mirounga leonina*, at King George Island, South Shetland Islands. Antarctic Science 72: 76–79.

[84] Daneri G, Carlini A (2002) Fish prey of southern elephant seals, *Mirounga leonina*, at King George Island. Polar Biology 25: 739–743.

[85] Polito MJ, Trivelpiece WZ, Karnovsky NJ, Ng E, Patterson WP, et al (2011) Integrating stomach content and stable isotope analyses to quantify the diets of pygoscelid penguins. PLoS ONE 6: 1–10. 10.1371/journal.pone.0026642 PMC3203888 22053199

[86] Tierney M, Southwell C, Emmerson LM, Hindell MA (2008) Evaluating and using stable-isotope analysis to infer diet composition and foraging ecology of Adelie penguins *Pygoscelis adeliae* . Marine Ecology Progress Series 355: 297–307.

[87] Quillfeldt P, McGill RAR, Furness RW (2005) Diet and foraging areas of Southern Ocean seabirds and their prey inferred from stable isotopes: review and case study of Wilson's storm-petrel. Marine Ecology Progress Series 295: 295–304.

[88] Hunt BPV, Hosie GW (2005) Zonal structure of zooplankton communities in the Southern Ocean South of Australia: results from a 2150 km continuous plankton recorder transect. Deep-Sea Research Part I: Oceanographic Research Papers 52: 1241–1271.

[89] Nicol S, Pauly T, Bindoff NL, Wright S, Thiele D, et al (2000) Ocean circulation off east Antarctica affects ecosystem structure and sea-ice extent. Nature 406: 504–507. 10952309 10.1038/35020053

[90] Worby AP, Massom RA, Allison I, Lytle VI, Heil P (1998) East Antarctic sea ice: A review of its structure, properties and drift. In: Jeffries MO, editor. Antarctic Sea Ice: Physical Processes, Interactions and Variability, Antarctic Research Series. Washington, D. C: 41–67.

[91] Lascara CM, Hofmann EE, Ross RM, Quetin LB (1999) Seasonal variability in the distribution of Antarctic krill, *Euphausia superba*, west of the Antarctic Peninsula. Deep-Sea Research Part I: Oceanographic Research Papers 46: 951–984.

[92] Croxall JP, Everson I, Kooyman GL, Ricketts C, Davis RW (1985) Fur seal diving behaviour in relation to vertical distribution of krill. Journal of Animal Ecology 54: 1–8.

[93] Bailleul Fdr, Authier M, Ducatez S, Roquet F, Charrassin J-Bt, et al (2010) Looking at the unseen: combining animal bio-logging and stable isotopes to reveal a shift in the ecological niche of a deep diving predator. Ecography 33: 1–10.

[94] Bentaleb I, Martin C, Vrac M, Mate B, Mayzaud P, et al (2011) Foraging ecology of Mediterranean fin whales in a changing environment elucidated by satellite tracking and baleen plate stable isotopes. Marine Ecology Progress Series 438: 285–302.

[95] Thiebot J, Cherel Y, Trathan P, Bost C (2011) Inter-population segregation in the wintering areas of macaroni penguins. Marine Ecology Progress Series 421: 279–290.

[96] Zbinden J, Bearhop S, Bradshaw P, Gill B, Margaritoulis D, et al (2011) Migratory dichotomy and associated phenotypic variation in marine turtles revealed by satellite tracking and stable isotope analysis. Marine Ecology Progress Series 421: 291–302.

[97] Greaves D, K., Hammill M, O., Eddington J, D., Pettipas D, Schreer J (2004) F (2004) Growth rate and shedding of vibrissae in the gray seal, *Halichoerus grypus*: a cautionary note for stable isotope diet analysis. Marine Mammal Science 20: 296–304.

[98] Zhao L, Schell D (2004) M (2004) Stable isotope ratios in harbor seal *Phoca vitulina* vibrissae: effects of growth patterns on ecological records. Marine Ecology Progress Series 281: 267–273.

[99] Polischuk SC, Hobson KA, Ramsay MA (2001) Use of stable-carbon and -nitrogen isotopes to assess weaning and fasting in female polar bears and their cubs. Canadian Journal of Zoology 79: 499.

[100] Habran S, Debier C, Crocker DE, Houser DS, Lepoint G, et al (2010) Assessment of gestation, lactation and fasting on stable isotope ratios in northern elephant seals (*Mirounga angustirostris*). Marine Mammal Science 26: 880–895.

[101] Stegall VK, Farley SD, Rea LD, Pitcher KW, Rye RO, et al (2008) Discrimination of carbon and nitrogen isotopes from milk to serum and vibrissae in Alaska Steller sea lions (*Eumetopias jubatus*). Canadian Journal of Zoology 86: 17–23.

[102] Ling JK (1966) The skin and hair of the southern elephant seal, *Mirounga leonina* (Linn.) I. The facial vibrissae. Australian Journal of Zoology 14: 855–866.

[103] Hindell MA, Lydersen C, Hop H, Kovacs KM (2012) Pre-partum diet of adult female bearded seals in years of contrasting ice conditions. PLoS ONE 7: e38307. 22693616 10.1371/journal.pone.0038307 PMC3365033

[104] Hindell MA, Bryden MM, Burton HR (1994) Early growth and milk-composition in southern elephant seals (*Mirounga leonina*). Australian Journal of Zoology 42: 723–732.

[105] Bornemann H, Kreyscher M, Ramdohr S, Martin T, Carlini A, et al (2000) Southern elephant seal movements and Antarctic sea ice. Antarctic Science 12: 3–15.

[106] Kareiva P, Odell G (1987) Swarms of predators exhibit “preytaxis” if individual predators use area-restricted search. The American Naturalist 130: 233–270.

[107] Sokolov S (2008) Chlorophyll blooms in the Antarctic Zone south of Australia and New Zealand in reference to the Antarctic Circumpolar Current fronts and sea ice forcing. Journal of Geophysical Research 113: C03022.

[108] Hindell MA, Bradshaw CJA, Sumner MD, Michael KJ, Burton HR (2003) Dispersal of female southern elephant seals and their prey consumption during the austral summer: relevance to management and oceanographic zones. Journal of Applied Ecology 40: 703–715.

[109] Boyd IL, Arnbom TA, Fedak MA (1994) Biomass and energy consumption of the South Georgia population of southern elephant seals In: Le Boeuf BJ, Laws RM, editors. Elephant seals: population ecology, behavior, and physiology. Berkley: University of California Press. 98–120.

[110] Rodhouse PG, Arnbom T, Fedak MA, Yeatman J, Murray AWA (1992) Cephalopod prey of the southern elephant seal *Mirounga leonina* L. Canadian Journal of Zoology. 70: 1007–1015.

[111] Newland C, Field IC, Nichols PD, Bradshaw CJA, Hindell MA (2009) Blubber fatty acid profiles indicate dietary resource partitioning between adult and juvenile southern elephant seals. Marine Ecology Progress Series 384: 303–312.

[112] Newsome SD, Bentall GB, Tinker MT, Oftedal OT, Ralls K, et al (2010) Variation in δ^1^ ^3^C and δ^1^ ^5^N diet - vibrissae trophic discrimination factors in a wild population of California sea otters. Ecological Applications 20: 1744–1752. 20945772 10.1890/09-1502.1

[113] Cherel Y, Pütz K, Hobson K (2002) Summer diet of king penguins (*Aptenodytes patagonicus*) at the Falkland Islands, southern Atlantic Ocean. Polar Biology 25: 898–906.

[114] Adams NJ, Klages NT (1987) Seasonal variation in the diet of the king penguin (*Aptenodytes patagonicus*) at sub-Antarctic Marion Island. Journal of Zoology 212: 303–324.

[115] Cherel Y, Hobson KA, Guinet C, Vanpe C (2007) Stable isotopes document seasonal changes in trophic niches and winter foraging individual specialization in diving predators from the Southern Ocean. Journal of Animal Ecology 76: 826–836. 17584388 10.1111/j.1365-2656.2007.01238.x

[116] Wienecke B, Robertson G (2006) Comparison of foraging strategies of incubating king penguins *Aptenodytes patagonicus* from Macquarie and Heard islands. Polar Biology 29: 424–438.

[117] Roper CFE (1969) Systematics and zoogeography of the worldwide bathypelagic squid *Bathyteuthis* (Cephalopoda: Oegopsida). Bulletin of the United States National Museum 291: 1–210.

[118] Hopkins TL, Torres JJ (1989) Midwater food web in the vicinity of a marginal ice zone in the western Weddell Sea. Deep-Sea Research 36: 543–560.

[119] Mayzuad P, Farber-Lorda J, Corre MC (1985) Aspects of the nutritional metabolism of two euphausiids: *Euphausia superba* and *Thysanoessa macrura*. In: Siegfried WR, Condy PR, Laws RM, editors. Antarctic nutrient cycles and food webs. Berlin: Springer. 330–338.

[120] Phleger CF, Nelson MM, Mooney BD, Nichols PD (2002) Interannual and between species comparison of the lipids, fatty acids and sterols of Antarctic krill from the US AMLR Elephant Island survey area. Comparative Biochemistry and Physiology Part B: Biochemistry and Molecular Biology 131: 733–747. 10.1016/s1096-4959(02)00021-0 11923086

[121] Croxall JP, Reid K, Prince PA (1999) Diet, provisioning and productivity responses of marine predators to differences in availability of Antarctic krill. Marine Ecology Progress Series 177: 115–131.

[122] Pakhomov EA, Perissinotto R, McQuaid CD (1994) Comparative structure of the macrozooplankton/micronekton communities of the Subtropical and Antarctic Polar Fronts Marine Ecology Progress Series. 111: 155–169.

[123] Wienecke B, Robertson G (2002) Foraging Areas of King Penguins from Macquarie Island in Relation to a Marine Protected Area. Environmental Management 29: 662–672. 12180180 10.1007/s00267-0015-1

[124] Hull C, Hindell M, Michael K (1997) Foraging zones of royal penguins during the breeding season, and their association with oceanographic features. Marine Ecology Progress Series 153: 217–228.

[125] Le Boeuf BJ, Morris PA, Blackwell SB, Crocker DE, Costa DP (1996) Diving behavior of juvenile northern elephant seals. Canadian Journal of Zoology 74: 1632–1644.

[126] Slip D, Hindell MA, Burton HR (1994) Diving behaviour of southern elephant seals from Macquarie Island: an overview. In: Le Boeuf BJ, Laws RM, editors. Elephant seals; population ecology, behaviour, and physiology. Berkeley: University of California Press. 66–84.

