# Supplementary material for: Spatially Explicit Estimates of Prey Consumption Reveal a New Krill Predator in the Southern Ocean
Source: PLoS One. 2014 Jan 24;9(1):e86452. doi: 10.1371/journal.pone.0086452 (PMC3905967; doi:10.1371/journal.pone.0086452)
Supplement: Figure S1 — Tracks overlaid with state estimates from the two-state first-difference correlated random walk switching (DCRWS) model. Tracks of 12 weaned southern elephant seals during their first foraging migration from Macquarie Island, colour coded by state estimates. Grey: transit locations; light blue, blue and green: Area Restricted Search (ARS) locations for S of SACCF-S, ACC to PF-S and PF zones, respectively. (PDF) [file pone.0086452.s001.pdf]

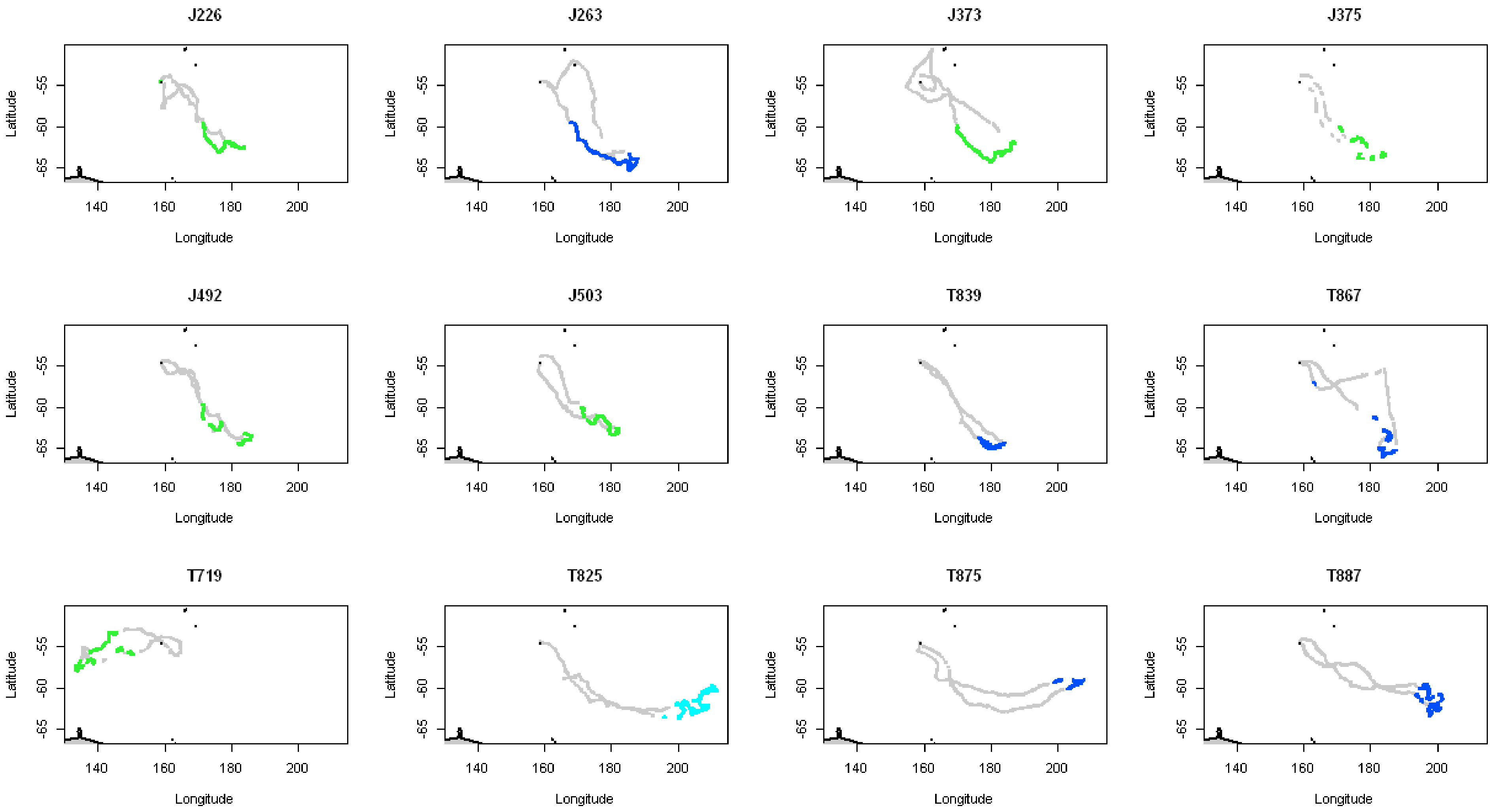

Figure S1. Tracks overlaid with state estimates from the two-state first-difference correlated random walk switching (DCRWS) model. Tracks of 12 weaned southern elephant seals during their first foraging migration from Macquarie Island, colour coded by state estimates. Grey: transit locations; light blue, blue and green: Area Restricted Search (ARS) locations for S of SACCF-S, ACC to PF-S and PF zones, respectively.
