# Supplementary material for: Spatially Explicit Estimates of Prey Consumption Reveal a New Krill Predator in the Southern Ocean
Source: PLoS One. 2014 Jan 24;9(1):e86452. doi: 10.1371/journal.pone.0086452 (PMC3905967; doi:10.1371/journal.pone.0086452)
Supplement: Table S1 — Stable carbon (δ13C) and nitrogen (δ15N) values of various marine organisms from the Southern Ocean. (PDF) [file pone.0086452.s002.pdf]

Table S1. Stable carbon ($\delta^{13}C$) and nitrogen ($\delta^{15}N$) values of various marine organisms from the Southern Ocean.

Species	Location	Tissue	n	$\delta^{13}C$ (‰)		$\delta^{15}N$ (‰)		Source
**Mid latitude waters (<55°S)**								
Euphausiids								
Euphausia frigida	Weddell Sea (49˚S, 20˚E)	Whole individuals	4	-24.3	± 0.9	4.9	± 0.3	Schmidt et al. (2004)
Fish								
Krefftichthys anderssoni	Kerguelen (49°21'S, 70°18'E)	White muscle	12	-22.3	± 0.2	7.6	± 0.2	Cherel et al. (2008)
Electrona antarctica	Kerguelen (49°21'S, 70°18'E)	White muscle	10	-22.2	± 0.9	8.0	± 0.5	This study
Gymnoscopelus fraseri	Kerguelen (49°21'S, 70°18'E)	White muscle	7	-22.5	± 1.1	8.0	± 0.5	This study
Gymnoscopelus nicholsi	Kerguelen (49°21'S, 70°18'E)	White muscle	10	-22.7	± 0.5	9.7	± 0.7	This study
Protomyctophum tenisoni	Kerguelen (49°21'S, 70°18'E)	White muscle	5	-22.1	± 1.0	6.4	± 0.3	This study
Gymnoscopelus piablis	Kerguelen (49°21'S, 70°18'E)	White muscle	2	-20.4	± 0.3	7.8	± 0.1	This study
Squid								
Histioteuthis eltaninae (LRL: 2.2±0.2 mm)	Macquarie Island (54˚30'S, 158˚57'E)	Beaks (corrected)	26	-21.2	± 0.6	7.8	± 1.9	Hughes (unpubl. data)
Martialia hyadesi (LRL: 6.5± 0.3 mm)	Macquarie Island (54˚30'S, 158˚57'E)	Beaks (corrected)	17	-21.6	± 1.2	6.6	± 1.5	Hughes (unpubl. data)
**High latitude waters (>60°S)**								
Amphipod								
Themisto gaudichaudii	Dumont d'Urville Sea (65°30'S, 143°E)	Whole individuals	7	-26.0	± 0.5	5.4	± 0.4	This study
Euphausiids								
Euphausia superba	Adélie Land (66°S, 136°E)	Whole individuals	12	-25.8	± 0.4	5.5	± 0.4	Cherel (2008)
Euphausia triacantha	Dumont d'Urville Sea (63°S, 140°E)	Whole individuals	10	-23.6	± 0.5	6.3	± 0.6	This study
Fish								
Electrona antarctica	Dumont d'Urville Sea (62°S, 140°E)	White muscle	9	-23.2	± 0.4	8.3	± 0.4	This study
Squid								
Psychrotheuthis glacialis	Northern Ross Sea (65°S, 180°E)	Mantle	20	-25.3	± 0.6	7.9	± 0.6	Bury et al.(2008)
Galiteuthis glacialis	Northern Ross Sea (65°S, 180°E)	Mantle	3	-24.7	± 0.9	8.1	± 0.4	Bury et al.(2008)
Kondakovia longimana	Northern Ross Sea (65°S, 180°E)	Mantle	20	-25.1	± 0.6	7.6	± 0.6	Bury et al.(2008)
Bathyteuthis abysicola	Dumont d'Urville Sea (63°S, 140°E)	Mantle	2	-24.2	± 0.3	8.0	± 0.3	This study
Psychrotheuthis glacialis	Dumont d'Urville Sea (65°30'S, 140°E)	Mantle	3	-25.9	± 0.2	10.0	± 0.5	This study

Beaks (corrected) = Squid beak isotopic values presented are corrected for reduced $\delta^{15}N$ enrichment due to chitin. See Materials and Methods for details.
LRL = Lower Rostral Length of squid beaks
